# Mouse α-Defensins: Structural and Functional Analysis of the 17 Cryptdin Isoforms Identified from a Single Jejunal Crypt

**DOI:** 10.1128/iai.00361-22

**Published:** 2022-12-06

**Authors:** Qingxia Wang, Yilin Yang, Gan Luo, Yang Zhou, William D. Tolbert, Marzena Pazgier, Chongbing Liao, Wuyuan Lu

**Affiliations:** a Key Laboratory of Medical Molecular Virology (MOE/NHC/CAMS), School of Basic Medical Science, and Shanghai Institute of Infectious Disease and Biosecurity, Fudan University, Shanghai, China; b Infectious Disease Division, Department of Medicine, Uniformed Services University of the Health Sciences, Bethesda, Maryland, USA; University of California—Davis

**Keywords:** defensins, cryptdins, antimicrobial peptides, host defense peptides, virtual colony count, structure-to-activity, structure-function

## Abstract

Mouse α-defensins, better known as cryptdins, are host protective antimicrobial peptides produced in the intestinal crypt by Paneth cells. To date, more than 20 cryptdin mRNAs have been identified from mouse small intestine, of which the first six cryptdins (Crp1 to Crp6) have been isolated and characterized at the peptide level. We quantified bactericidal activities against Escherichia coli and Staphylococcus aureus of the 17 cryptdin isoforms identified by Ouellette and colleagues from a single jejunal crypt (A. J. Ouellette et al., Infect Immun 62:5040–5047, 1994), along with linearized analogs of Crp1, Crp4, and Crp14. In addition, we analyzed the most potent and weakest cryptdins in the panel with respect to their ability to self-associate in solution. Finally, we solved, for the first time, the high-resolution crystal structure of a cryptdin, Crp14, and performed molecular dynamics simulation on Crp14 and a hypothetical mutant, T14K-Crp14. Our results indicate that mutational effects are highly dependent on cryptdin sequence, residue position, and bacterial strain. Crp14 adopts a disulfide-stabilized, three-stranded β-sheet core structure and forms a noncanonical dimer stabilized by asymmetrical interactions between the two β1 strands in parallel. The killing of E. coli by cryptdins is generally independent of their tertiary and quaternary structures that are important for the killing of S. aureus, which is indicative of two distinct mechanisms of action. Importantly, sequence variations impact the bactericidal activity of cryptdins by influencing their ability to self-associate in solution. This study expands our current understanding of how cryptdins function at the molecular level.

## INTRODUCTION

Antimicrobial peptides (AMPs) serve as ancient weapons of defense against bacteria, fungi, and viruses and are essential to innate immunity in complex multicellular organisms from insects to humans (1–8). Defensins, a term coined in 1985 by Robert I. Lehrer of UCLA to describe human neutrophil peptides 1, 2, and 3 (9, 10), represent a major family of AMPs found in mammals. Mammalian defensins of 2 to 5 kDa in size are rich in Arg and/or Lys residues with three intramolecular disulfide bonds and are classified into α, β, and θ subfamilies according to their disulfide topology and sites of expression. Six human α-defensins have been identified thus far, including four primarily from neutrophils, also known as human neutrophil peptides 1, 2, 3, and 4 or HNP1-4 (9–13), and human defensins 5 and 6 or HD5-6 mainly from intestinal Paneth cells (14, 15). The connectivity of the six invariant Cys residues in the six human α-defensins is Cys1-Cys6, Cys2-Cys4, and Cys3-Cys5 (16), whereas a Cys1-Cys5, Cys2-Cys4, and Cys3-Cys6 topology characterizes products of the significantly higher number of human β-defensin genes found predominantly in epithelial cells and tissues (17, 18). Despite the differences in amino acid sequence and disulfide topology, both human α- and β-defensins are similar at the tertiary structural level, adopting a disulfide-stabilized, three-stranded β-sheet core structure (19–25). The θ-defensins, expressed only in nonhuman primates, are macrocyclic peptides of 18-amino-acid residues with three disulfide bonds arranged in a ladder pattern (26, 27).

Mouse leukocytes do not express α-defensins (28), but their intestinal Paneth cells do. Mouse α-defensins, better known as cryptdins, are packaged in apically oriented granules of Paneth cells and are secreted within minutes into the crypt lumen in response to bacteria, bacterial antigens, cholinergic stimuli or certain nutrients at an estimated local concentration of 15 to 100 mg/mL (29–33). Cryptdins are initially synthesized as inactive precursors, i.e., procryptdins, which are proteolytically processed to their active mature forms by matrix metalloproteinase 7 (MMP-7 or matrilysin) (34, 35). Ouellette and colleagues identified mRNAs encoding more than 20 cryptdin isoforms in mouse small intestine (29, 36), of which 17 (Crp1 to Crp17) were cloned from a cDNA library generated from a single jejunal crypt of an outbred Swiss mouse (37). The inbred C57BL/6 mice lack the genes for Crp1, Crp2, Crp4, and Crp6, and express instead high levels of Crp20, Crp21, Crp23, Crp24, and Crp27 in addition to Crp3 and Crp5, indicative of strain-specific polymorphisms in cryptdin expression (36). Despite their diversity and abundance at the genomic level, only the first six cryptdins (Crp1 to Crp6) isolated from small intestines of adult mice have been characterized at the peptide level, whereas the remaining 11 cryptdins (Crp7 to Crp17) are expressed below the level of biochemical detection/purification (37).

Both human and mouse α-defensins of enteric origin are host protective against intestinal pathogens. Bevins and colleagues demonstrated that HD5- and HD6-transgenic mice are largely resistant to infection by virulent Salmonella enterica serovar Typhimurium, where HD5 directly kills the bacterium and HD6 forms a high-ordered “nanonet” structure to entrap it (38, 39). In contrast, MMP7-null mice are susceptible to Salmonella infection because they lack active cryptdins in Paneth cell granules and accumulate only nonbactericidal procryptdins in their small intestines (34, 40). While the host protective role is well established for enteric α-defensins, functional anomalies do exist in certain biological settings. HD5 and HD6 (41, 42) and Crp3 (43) have been reported to promote HIV infection *in vitro* by acting on the virion to enhance its attachment to target cells or facilitate viral entry. HD5 has also been shown to promote infection by certain serotypes of human adenovirus *in vitro* by enhancing viral attachment to target cells independent of receptor binding (44), and to augment their transgene expression and immunogenicity as vaccine vectors in mice (45). Wilson et al. used a mouse enteroid (enteric organoid) model to demonstrate that cryptdins enhance viral infection *ex vivo* of an enteric mouse pathogen, mouse adenovirus 2 (46). More recently, Xu et al. reported an unexpected finding that HD5 promotes *Shigella* infection *in vitro*, *in vivo*, and *ex vivo* by enhancing bacterial adhesion to epithelial cells and tissues, leading to the activation of the type III secretion system of *Shigella*, an obligate step toward bacterial invasion (47, 48). Growing evidence appears to suggest that defensins can play pathogenic roles in certain biological settings against the backdrop of their protective functions in antiviral and antibacterial immunity (49).

**FIG 1:**
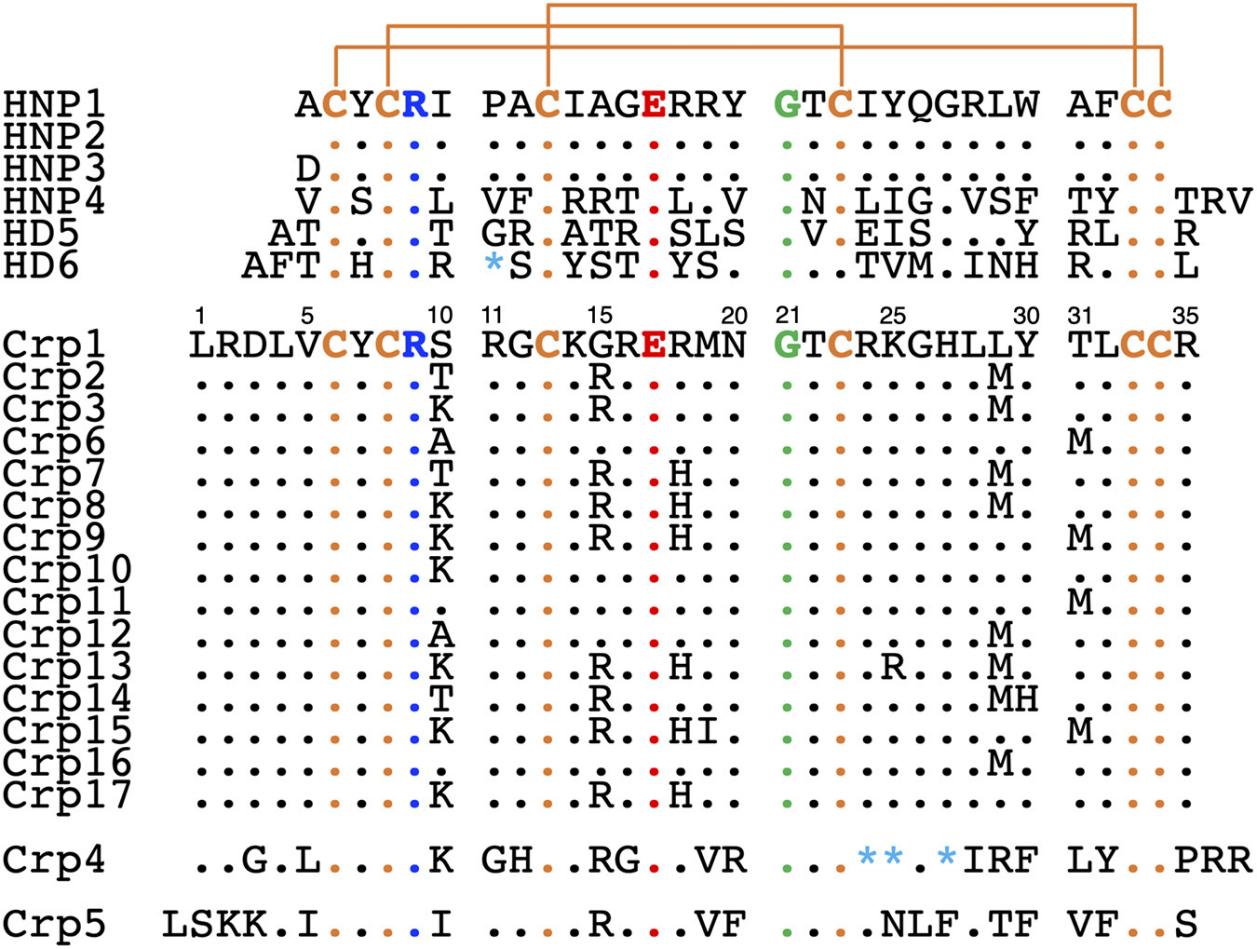
Amino acid sequences of the six human α-defensins (HNP1 to HNP4, HD5, and HD6) and of the 17 mouse cryptdin isoforms (Crp1 to Crp17). The conserved residues are colored, including six Cys residues (orange), one Arg (blue), one Glu (red), and one Gly (green). Asterisks in cyan denote amino acid deletions in the sequences. Crp4 and Crp5 are listed at the bottom to highlight the three subclasses of cryptdins, i.e., Crp1-like, Crp4, and Crp5.

While a large body of literature exists on the structure, function, and mechanisms of action of human defensins (16, 50), few studies focus on cryptdins despite the wealth of sequence information available. Based on sequence variability, the 17 cryptdins identified from a single jejunal crypt can be grouped into three subclasses: Crp1-like, Crp4, and Crp5, of which Crp1-like peptides are highly similar and form the largest group of cryptdins comprising Crp1 to Crp3 and Crp6 to Crp17 (29, 37) (Fig. 1). Many of the structure-activity relationship (SAR) studies on cryptdins have thus far been limited to Crp4 (51–60), which has the most potent bactericidal activities *in vitro* of Crp1 to Crp6 (29, 37). In fact, the nuclear magnetic resonance (NMR) solution structure of Crp4 is the only structure of any cryptdin determined to date (56, 61). Here, we describe a SAR study of all the 17 cryptdin isoforms with respect to their bactericidal activities against both E. coli and S. aureus and determine the first X-ray crystal structure of any cryptdin—Crp14—from the Crp1-like subclass.

## RESULTS

### Oxidative folding of chemically synthesized cryptdin peptides.

As is the case with all human α-defensins (62, 63), cryptdins can be chemically synthesized and oxidatively folded to their native structures stabilized by three disulfides (64, 65). All folded cryptdins displayed a reduction in mass of 6 Da compared with their unfolded counterparts, indicative of the formation of three disulfide bonds (see Fig. S1 to S3 in the supplemental material). For comparison, several linear analogs of cryptdins, in which six Cys residues were all replaced by Ala, were also prepared, including Crp1, Crp4, and Crp14 (see Fig. S4).

### Analysis of the X-ray crystal structure of Crp14.

**FIG 2:**
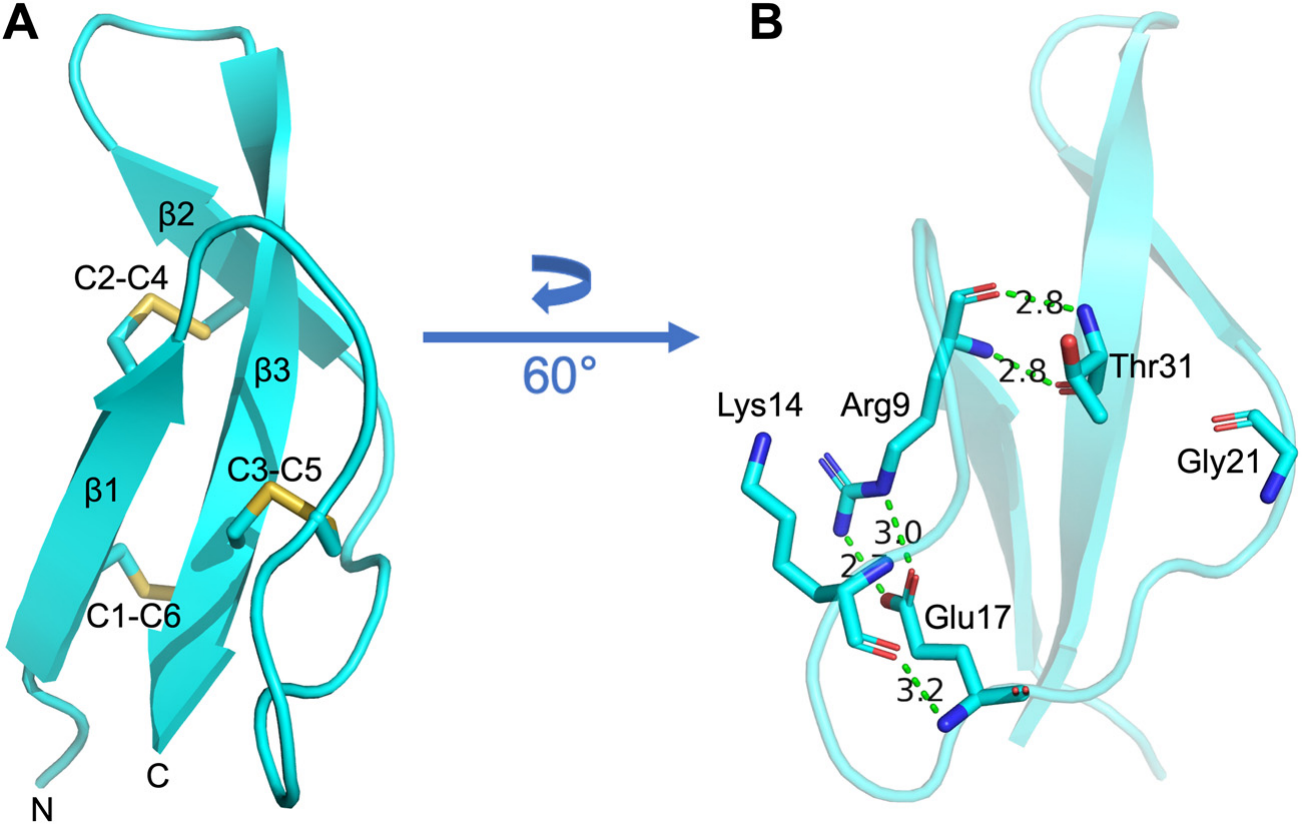
Crystal structure of Crp14. (A) A three-stranded antiparallel β-sheet stabilized by three intramolecular disulfide bonds (Cys1-Cys6, Cys2-Cys4, and Cys3-Cys5 in sequential numbering) structurally defines Crp14 and all known mammalian α-defensins. An invariant Gly residue (Gly21 in Crp14) in the β-bulge connecting the β2 and β3 strands propagates a dramatic twisting of β3 in relation to β2 to permit correct folding. (B) The loop connecting the β1 and β2 strands is stabilized by, in addition to disulfide bonding (not shown for clarity), an intricate H-bond network involving a fully conserved salt bridge between Arg9 and Glu17, two reciprocal main-chain/main-chain H-bonds between Arg9 and Thr31, and two additional H-bonds between Lys14 and Glu17. The image was prepared with the PyMOL Molecular Graphics System (v2.0; Schrödinger, LLC).

We conducted a preliminary screening for crystallization conditions with several cryptdin peptides and obtained diffractable crystals for Crp5 and Crp14. However, only the structure of Crp14 has been determined by X-ray crystallography to a high resolution of 1.67 Å (see Table S1). The first two amino acid residues Leu1 and Arg2 of Crp14 are missing from its electron density map presumably due to disorder at the N terminus (Crp1 or Crp14 numbering, Fig. 1; see also Fig. S5 in the supplemental material). As is the case with all known defensin structures, the core tertiary structure of Crp14 is a three-stranded antiparallel β-sheet comprising strand β1 (residues 5 to 9), strand β2 (residues 18 to 25), and strand β3 (residues 28 to 34), stabilized globally by three intramolecular disulfide bonds in the canonical “1-6, 2-4, 3-5” topology, i.e., Cys6-Cys34, Cys8-Cys23, and Cys13-Cys33 (Fig. 2A). Arg9 and Glu17 form a salt bridge that is conserved in all mammalian α-defensins. This salt bridge rigidifies the first loop connecting β1-β2 (56, 66) and is additionally stabilized by a Lys14 O accepting H-bond from Glu17 N and a Lys14 N donating H-bond to Glu17 O^ε1^ (Fig. 2B). Of note, Arg9 also forms two backbone-backbone H-bonds with Thr31, likely contributing to the stability of Crp14 core structure.

**FIG 3:**
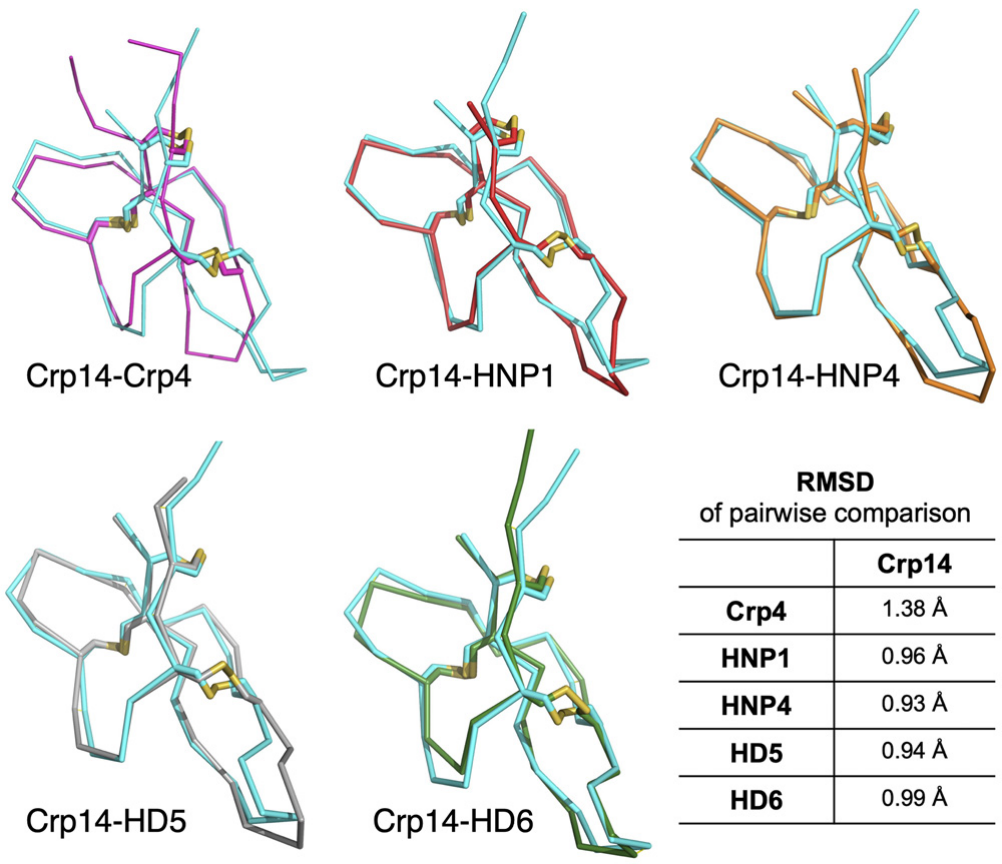
Structural comparisons of Crp14 (cyan) with other defensins. Monomers of Crp4 (magenta, PDB code 2GW9), HNP1 (red, PDB code 3GNY), HNP4 (orange, PDB code 1ZMM), HD5 (gray, PDB code 1ZMP), and HD6 (green, PDB code 1ZMQ) were used for superposition. The RMSDs following alignment by Cα atoms were analyzed using PDBeFold (https://www.ebi.ac.uk/msd-srv/ssm/). The image was prepared with the PyMOL Molecular Graphics System (v2.0; Schrödinger, LLC).

Despite the rather low sequence identity Crp14 shares with Crp4 (62%), HNP1 (42%), HNP4 (43%), HD5 (47%), and HD6 (39%), their tertiary structures are highly similar. When Crp14 monomer (chain A) is superimposed with that of Crp4, HNP1, HNP4, HD5, or HD6, root mean square deviations (RMSDs) of the Cα atoms from their superpositions are 1.38, 0.96, 0.93, 0.94, and 0.99 Å, respectively (Fig. 3). Notably, Crp14 differs more from its mouse cousin Crp4 (RMSD 1.38 Å) than from its human counterparts, underscoring the structural impact of the three-residue deletion that is unique to Crp4 (Fig. 1) in the second loop connecting the β2 and β3 strands. In addition, the “excessive” disorder within the N terminus of the NMR solution structure of Crp4 (56, 61) may also contribute to the high RMSD value compared to Crp14 and Crp4.

**FIG 4:**
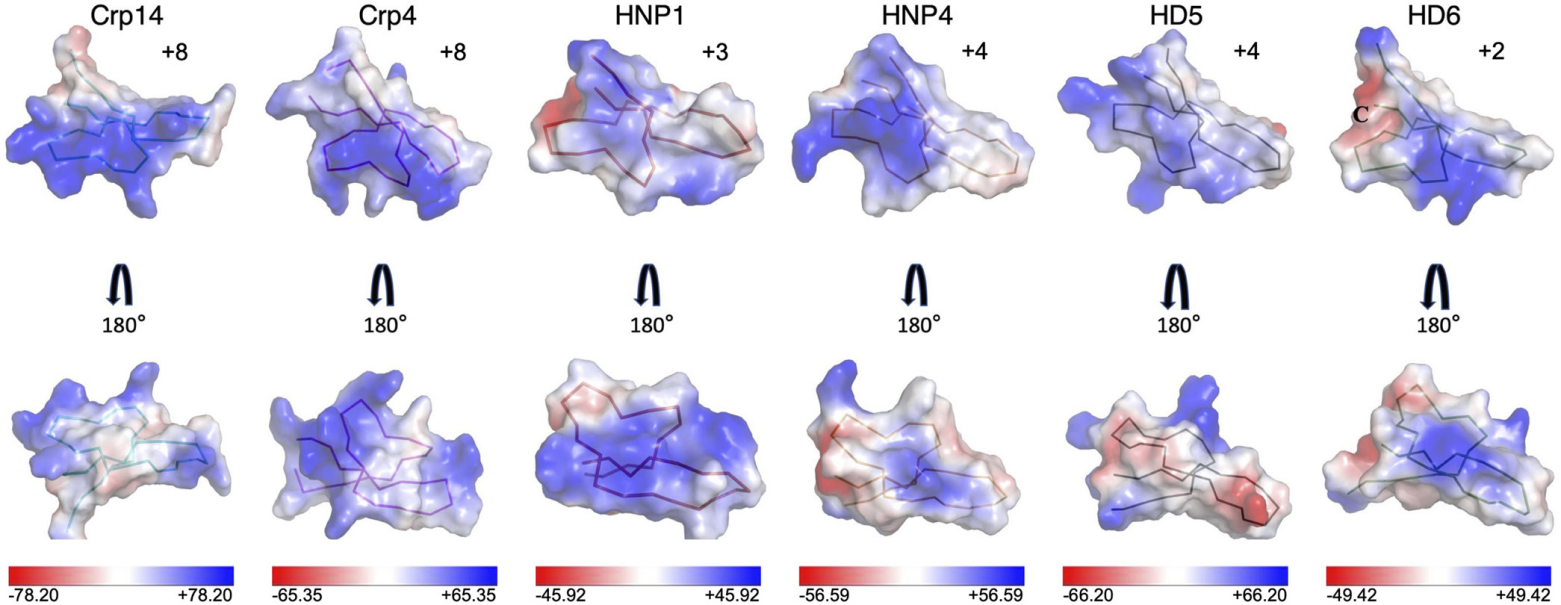
Charge distribution on the surfaces of Crp14 and other defensins—Crp4, HNP1, HNP4, HD5, and HD6. The basic regions of the defensins are colored blue, whereas the acidic regions are colored red. For comparison, the net charge of each defensin at pH 7.4 is also shown as +8 for Crp14, +8 for Crp4, +3 for HNP1, +4 for HNP4, +4 for HD5, and +2 for HD6. The image was prepared with the PyMOL Molecular Graphics System (v2.0; Schrödinger, LLC).

As shown in the electrostatic potential map (Fig. 4), Crp14 is clearly amphiphilic with a cluster of positively charged residues well segregated from a large hydrophobic surface. The cationicity of cryptdins is dominated by a consensus sequence located in the loop connecting the β1 and β2 strands, i.e., Lys14-Arg15-Arg16-Glu17-Arg/His18. In fact, the invariant Lys14 in cryptdins shields the negatively charged Glu17 from bulk medium by stacking its side chain against the fully conserved salt bridge between Glu17 and Arg9 (Fig. 2B). In contrast, human α-defensins are significantly less cationic than Crp14 (Fig. 1), as a result, less amphiphilic (Fig. 4).

**FIG 5:**
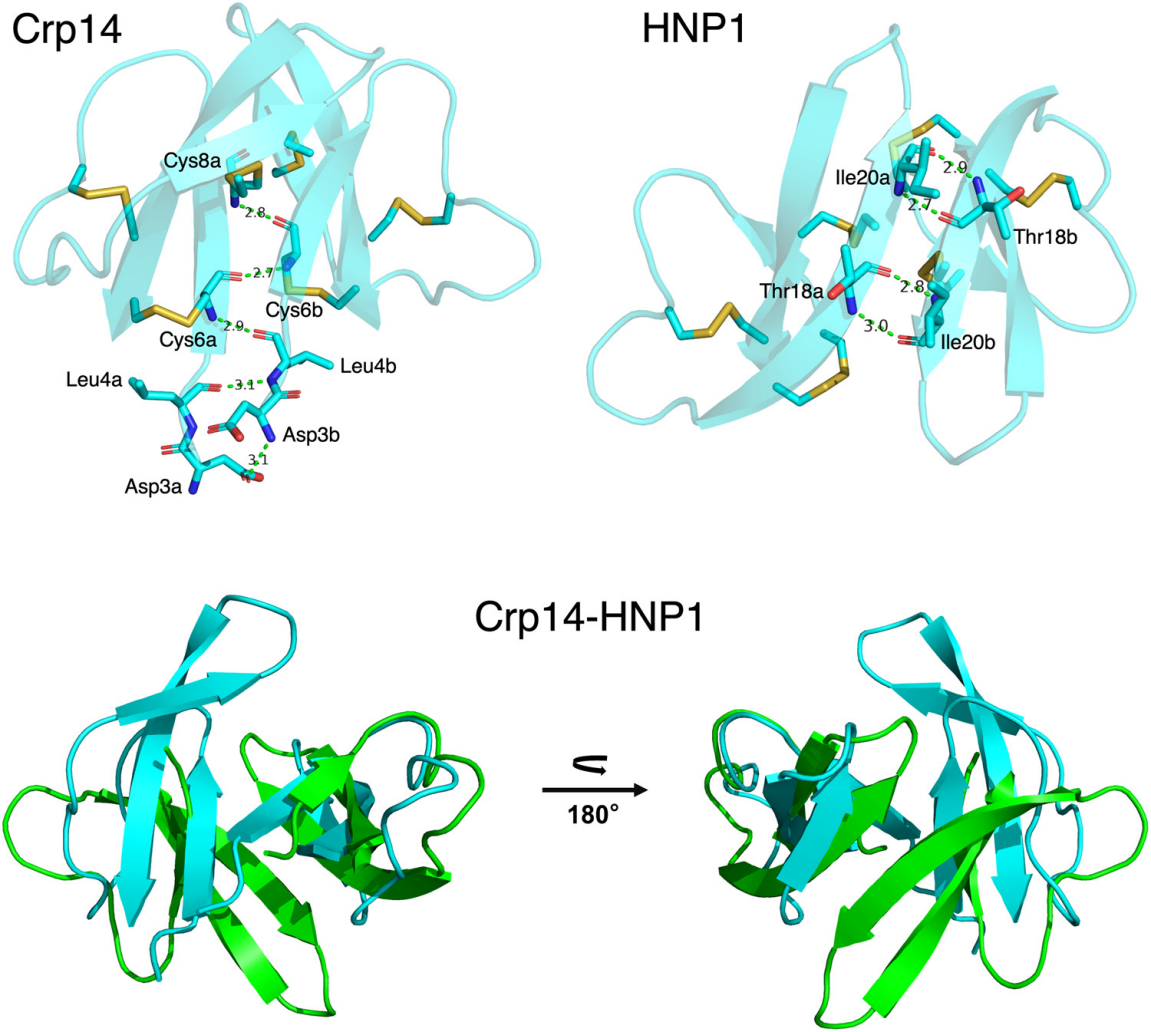
Two distinctive modes of defensin dimerization seen in the crystal structures of Crp14 and HNP1. Crp14 dimerizes asymmetrically via the two β1 strands in parallel, participated by the N-terminal residues Asp3, Leu4, Cys6, and Cys8 forming five H-bonds (top left). The canonical mode of (symmetric) dimerization, conserved in all other mammalian α-defensins with known crystal structures, is depicted by the crystal structure of HNP1 (PDB code 3gny) (76), where the dimer interface is formed by the two β2 strands in antiparallel and stabilized by four reciprocal backbone H-bonds contributed by Thr18 and Ile20 (top right). Superposition of the Crp14 (cyan) and HNP1 (green) dimers aligned on the Cα atoms of their A chains reveals dramatic changes in the topology of their B chains (bottom). For clarity, the three disulfide bonds are not shown. The image was prepared with the PyMOL Molecular Graphics System (v2.0; Schrödinger, LLC).

Crp14 crystallized in space group P6_5_22 with two Crp14 molecules in the asymmetric unit that form an apparent dimer that is different from the dimers of other α-defensins with known crystal structures. As shown in Fig. 5, Crp14 dimerization is mediated primarily by interactions between the two β1 strands in parallel, in contrast to the dimers of other known α-defensins that dimerize via the interactions of the two β2 strands in antiparallel. Four asymmetric main chain-main chain H-bonds form the basis of the dimer interface, i.e., Leu4a O-Leu4b N, Cys6a N-Leu4b O, Cys6a O-Cys6b N, and Cys8a N-Cys6b O. In addition, Asp3b N donates an H-bond to Asp3a O^δ1^ and the side chain of Tyr7 packs against neighboring disulfide bonds in both copies. In contrast, in the prototypic α-defensin HNP1 (Fig. 5), Thr18 and Ile20 of one monomer each reciprocally donate an H-bond to and accept an H-bond from Ile20 and Thr18 of the opposing monomer, respectively, to form four stabilizing symmetric backbone H-bonds. This reorients chain B of Crp14 in relation to that of HNP1 when the two dimers are superimposed on their A chains, to yield two dramatically different quaternary structures (Fig. 5). However, the total buried surface area (BSA) for both dimers is similar, 724 Å^2^ for the Crp14 dimer compared to 744 Å^2^ for HNP1. Crystal packing in the higher symmetry Crp14 space group suggests other possible molecular interfaces that could be used by Crp14 monomers to associate, but none are as extensive as that of the one in the asymmetric unit. The side chains Arg11 and Arg18 of adjacent monomers in one interface and the side chains of Leu28 and Met29 in adjacent monomers of another constitute the interfaces with the next highest BSAs, 715 Å^2^ for the former and 591 Å^2^ for the latter, but the high BSA values in both cases are largely due to the contribution of side chains at the interface. Other crystal contact interfaces have significantly lower total BSAs which suggests that the dimer in the asymmetric unit is the most physiologically relevant one. However, we cannot unambiguously exclude the possibility that at lower protein concentrations in solution this dimer may be less prevalent or not formed at all.

### Highly variable antibacterial activities of cryptdins.

**FIG 6:**
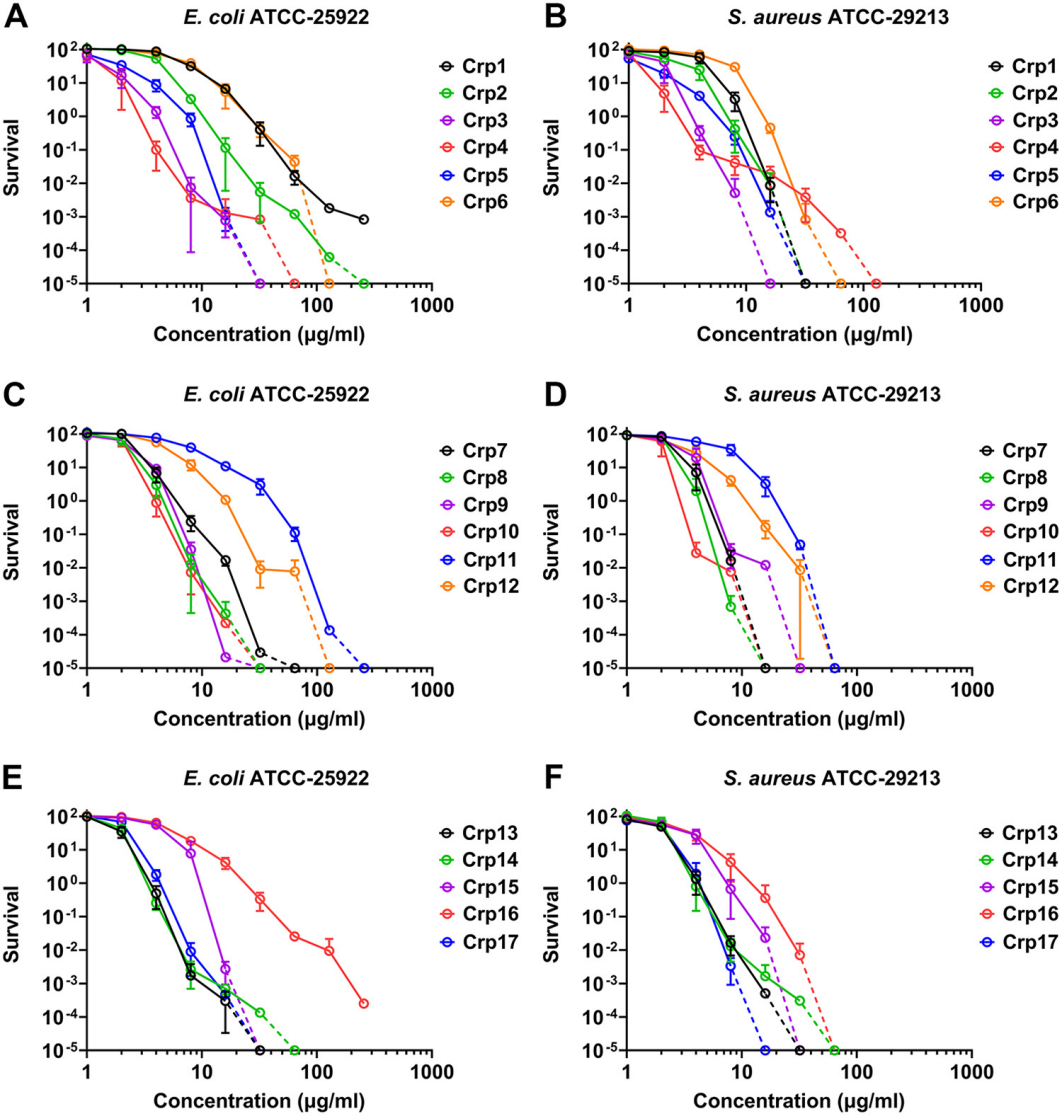
Virtual colony count survival curves of E. coli and *S aureus* exposed to cryptdins. Survival curves of E. coli ATCC 25922 and of S. aureus ATCC 29213 exposed to Crp1 to Crp6 (A and B), Crp7 to Crp12 (C and D), and Crp13 to Crp17 (E and F). Each curve is the mean of triplicate experiments. Note that zero survival cannot be plotted on a logarithmic scale. To illustrate complete killing, some curves are extended with dotted lines to cross the *x* axis at the concentration where zero survival is reported.

All cryptdins were subjected to a virtual colony count assay (vCC) we have previously developed (67), in which E. coli ATCC 25922 and S. aureus ATCC 29213 are exposed to a 2-fold serial dilution of cryptdin for 2 h in 10 mM sodium phosphate buffer (pH 7.4), before bacterial growth in the presence of Mueller-Hinton broth is monitored spectrophotometrically at 650 nm for a period of 12 h. The dose-dependent killing of E. coli and S. aureus by all 17 cryptdins is shown as bacterial survival curves in Fig. 6. The virtual lethal doses (vLDs) of cryptdins at 50%, 10%, 1% and 0.1% bacterial survival (or the vLD50, vLD90, vLD99, and vLD99.9 values) are tabulated in Table 1. The bactericidal activity of the Crp1 subclass of cryptdins is highly variable despite a high degree of sequence identity, with Crp1, Crp6, Crp11, and Crp16 being the weakest against both strains and Crp3, Crp8 to Crp10, Crp13, Crp14, and Crp17 being the strongest. The vLD50, vLD90, vLD99 and vLD99.9 values for Crp11 to E. coli differ from those for Crp14 by 3.4-, 7.0-, 11.8-, and 12.7-fold, respectively; for comparison, the vLD values for Crp11 to S. aureus are 3.1-, 5.0-, 6.8-, and 8.1-fold versus those for Crp10, respectively. Overall, S. aureus appears more susceptible to cryptdin killing than E. coli.

**TABLE 1:** Antibacterial virtual lethal doses of cryptdin^*a*^

Defensin	Mean vLD (μg/mL) ± SD							
	*E. coli* ATCC 25922				*S. aureus* ATCC 29213			
	vLD50	vLD90	vLD99	vLD99.9	vLD50	vLD90	vLD99	vLD99.9
Crp1	5.9 ± 0.3	13.4 ± 0.6	25.4 ± 2.3	41.9 ± 4.8	3.9 ± 0.6	6.1 ± 0.5	9.1 ± 0.3	11.9 ± 0.5
L-Crp1	2.3 ± 0.0	3.1 ± 0.1	LOD	LOD	4.2 ± 0.2	7.2 ± 0.3	11.5 ± 0.6	17.1 ± 1.2
Crp2	4.1 ± 0.1	6.1 ± 0.1	10.1 ± 0.5	15.9 ± 2.3	2.3 ± 0.4	4.6 ± 0.4	6.7 ± 0.8	9.8 ± 1.8
Crp3	1.1 ± 0.2	2.2 ± 0.4	4.1 ± 0.1	5.6 ± 0.3	1.5 ± 0.5	2.4 ± 0.2	3.4 ± 0.2	4.8 ± 0.5
Crp4	1.2 ± 0.2	1.9 ± 0.3	2.8 ± 0.3	3.8 ± 0.5	1.1 ± 0.1	1.7 ± 0.2	2.6 ± 0.3	4.2 ± 0.7
L-Crp4	2.0 ± 0.2	2.3 ± 0.1	2.8 ± 0.2	3.4 ± 0.4	2.0 ± 0.0	2.4 ± 0.1	2.9 ± 0.1	3.5 ± 0.1
Crp5	1.4 ± 0.2	3.7 ± 0.4	7.5 ± 0.7	9.9 ± 0.5	1.1 ± 0.0	2.7 ± 0.1	5.7 ± 0.3	9.0 ± 0.1
Crp6	6.3 ± 0.9	12.9 ± 2.0	24.6 ± 2.6	51.2 ± 3.6	5.4 ± 0.5	9.6 ± 0.3	14.0 ± 0.6	17.4 ± 1.8
Crp7	2.4 ± 0.1	3.6 ± 0.3	5.8 ± 0.5	9.8 ± 0.6	2.3 ± 0.1	3.6 ± 0.5	4.8 ± 0.5	6.3 ± 0.7
Crp8	2.1 ± 0.1	3.0 ± 0.2	4.5 ± 0.4	6.0 ± 0.7	2.2 ± 0.0	2.9 ± 0.3	3.9 ± 0.4	5.1 ± 0.1
Crp9	2.2 ± 0.2	3.8 ± 0.2	5.2 ± 0.2	6.9 ± 0.5	2.5 ± 0.5	3.8 ± 0.6	5.2 ± 0.3	6.8 ± 0.5
Crp10	2.1 ± 0.1	2.7 ± 0.0	3.9 ± 0.3	5.4 ± 0.5	1.8 ± 0.4	2.3 ± 0.2	2.8 ± 0.2	3.5 ± 0.3
Crp11	6.2 ± 0.3	16.9 ± 1.4	39.0 ± 3.1	58.2 ± 0.0	5.5 ± 1.5	11.5 ± 1.2	19.1 ± 0.9	28.4 ± 0.7
Crp12	4.1 ± 0.4	8.3 ± 0.8	16.2 ± 0.1	22.5 ± 0.9	2.6 ± 0.6	5.7 ± 0.6	10.7 ± 0.4	17.5 ± 2.0
Crp13	1.6 ± 0.2	2.4 ± 0.2	3.5 ± 0.3	4.7 ± 0.4	2.0 ± 0.1	2.7 ± 0.1	4.1 ± 0.3	5.9 ± 0.2
Crp14	1.8 ± 0.0	2.4 ± 0.0	3.3 ± 0.1	4.6 ± 0.2	2.0 ± 0.2	2.7 ± 0.2	3.8 ± 0.4	5.5 ± 0.5
L-Crp14	2.1 ± 0.0	2.5 ± 0.1	3.1 ± 0.3	3.9 ± 0.7	4.1 ± 0.5	6.5 ± 0.8	10.6 ± 1.1	16.6 ± 1.7
Crp15	4.2 ± 0.4	6.6 ± 1.3	9.1 ± 0.8	11.3 ± 0.8	2.4 ± 0.5	4.7 ± 0.2	7.3 ± 0.9	11.0 ± 2.0
Crp16	4.6 ± 0.4	10.7 ± 1.1	23.5 ± 2.5	41.0 ± 2.9	2.6 ± 0.4	5.8 ± 1.1	11.3 ± 3.1	17.0 ± 4.3
Crp17	2.1 ± 0.1	2.9 ± 0.1	4.3 ± 0.2	5.8 ± 0.4	2.0 ± 0.0	2.8 ± 0.3	4.0 ± 0.5	5.3 ± 0.6

a The antibacterial virtual lethal doses of cryptdin (vLD) at which 50, 90, 99, and 99.9% of E. coli or S. aureus input viable cells are killed. The data were obtained from three independent assays. LOD, limit of detection.

Crp4 and Crp5 share ~50% sequence identity, and the former is more active than the latter in the low concentration range which is apparent by the ~2-fold difference in their vLD values. However, the opposite is true at higher concentrations. In the low concentration range, Crp4 is also slightly more active than Crp3, Crp10, Crp13, and Crp14, making it the most active of all 17 cryptdins, as judged by their measured vLD values. Again, however, multiple cryptdins displayed stronger bactericidal activity at higher concentrations, where Crp4 did not eliminate bacterial growth as shown by its killing curves (red curves in Fig. 6A and B). For instance, while Crp4 at 32 μg/mL reduced E. coli survival by 5 orders of magnitude, the bacterium never recovered in the presence of Crp3 or Crp5 at the same concentration (Fig. 6A). Similarly, Crp4 at 64 μg/mL reduced S. aureus survival by more than 5 orders of magnitude, but Crp3 at 16 μg/mL and Crp1, Crp2, and Crp5 at 32 μg/mL completely killed it (Fig. 6B). Although it remains difficult to discern the SAR among the three subclasses of cryptdins due to their relatively low degrees of sequence identity, pairwise comparisons within the Crp1 subclass comprising the highly similar Crp1 to Crp3 and Crp6 to Crp17 peptides are not only feasible but also informative.

Of note, the published bactericidal data on cryptdins (lethal doses) were often obtained in an actual colony count assay and presented in the form of μg/mL or μM. The concentration range we used (0 to 256 μg/mL) in the *virtual* colony count assay is wider, allowing us to quantify the activity of weakly bactericidal peptides. Despite the methodological differences, the activity data generated from the two different assay systems are rather comparable. For example, Crp4 at 5 μg/mL reduced the survival in CFU/mL of E. coli ML35 and S. aureus 710a by ~3 ± 1 orders of magnitude (36, 54, 57, 60), similar to the vLD99.9 values of 3.8 and 4.2 μg/mL for E. coli ATCC 25922 and S. aureus ATCC 29213, respectively.

### Dissection of the SAR for the Crp1 subclass of cryptdins.

We made pairwise comparisons for cryptdins at all eight positions where variations occur, including 10, 15, 18, 19, 25, 29, 30, and 31 (Crp1 numbering, Fig. 1). Some positions have been grouped together for presentation due to the similar nature of their variations.

### Position 10.

**FIG 7:**
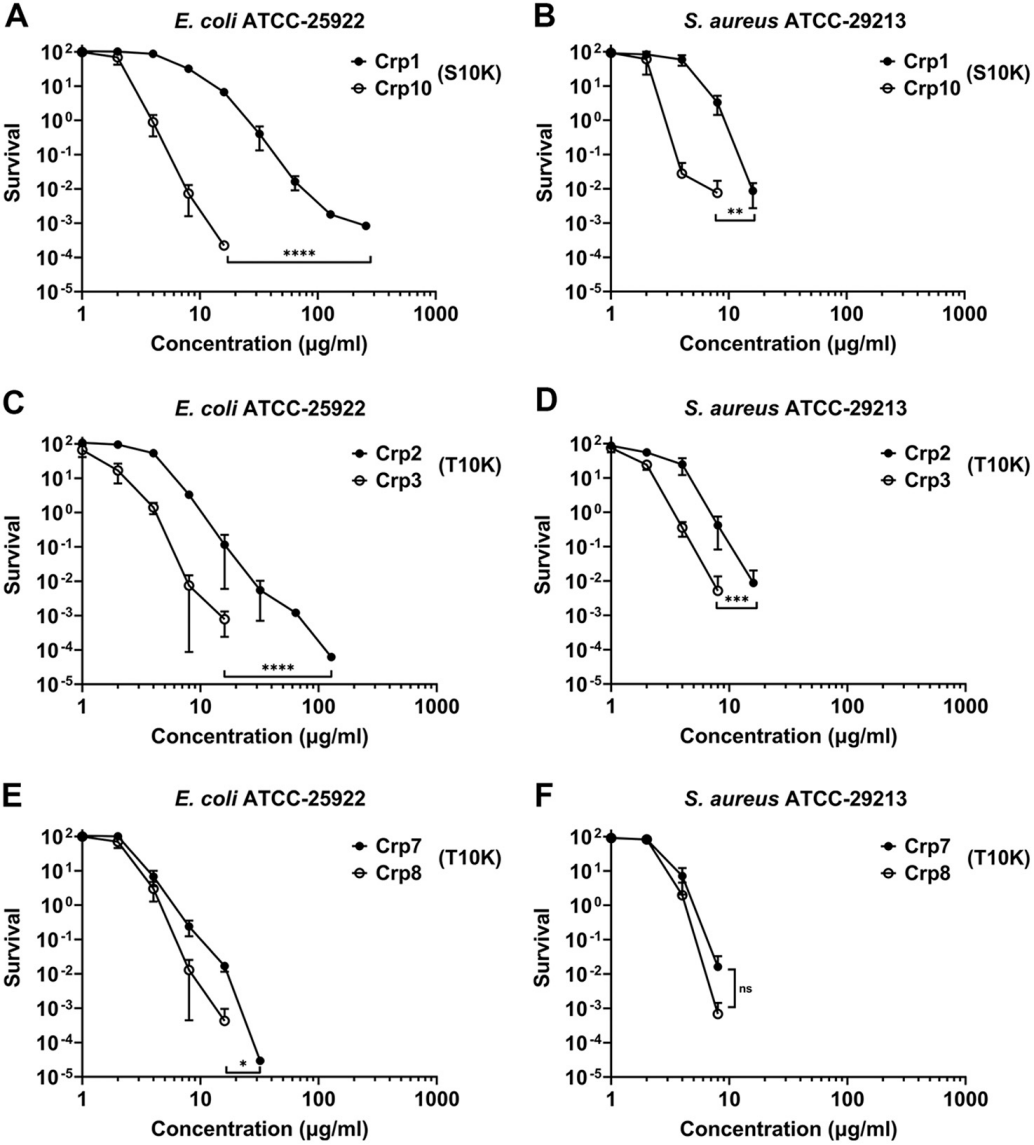
Pairwise comparisons of cryptdins in the killing of E. coli and S. aureus. Each curve is the mean of triplicate experiments. These killing curves of cryptdins are re-graphed from Fig. 6. Statistical significance was determined by using a two-way ANOVA test (*, *P* < 0.05; **, *P* < 0.01; ***, *P* < 0.001; ****, *P* < 0.0001).

Lys is the most frequent residue found at position 10 (in seven cryptdins), followed by Ser and Thr (3 each) and Ala (2). A single variation, S10K, converts Crp1 to Crp10, which is accompanied by a substantial increase in bactericidal activity against E. coli (a 3- to 8-fold reduction in vLD value) (Fig. 7A). The variation’s effect in the Crp1 or Crp10 killing of S. aureus is less pronounced though (Fig. 7B), corresponding to only a 2- to 3-fold reduction in vLD value. Similarly, the T10K variation needed to change Crp2 to Crp3 or Crp7 to Crp8 enhances bactericidal activity for both, but not dramatically. The vLD values for the Crp2/Crp3 pair differ by ~2-fold to both strains (Fig. 7C and D), and Crp8 is only ~20% more active than Crp7 (Fig. 7E and F).

A charge-neutral variation, A10S, differentiates Crp6 from Crp11 and Crp12 from Crp16. This variation is largely inconsequential except at high concentrations for the killing of E. coli, where the nonpolar residue Ala appears preferred over the polar residue Ser (see Fig. S6A and C). This preference is also evident in the killing of S. aureus by the Crp6/Crp11 pair (see Fig. S6B). The A10S variation increases the vLD99 value from 14.0 to 19.1 μg/mL and the vLD99.9 value from 17.4 to 28.4 μg/mL. On the other hand, Crp12 is virtually identical to Crp16 with respect to the killing of S. aureus (see Fig. S6D). Taken together, these results suggest that while the cationic residue Lys at position 10 is important in cryptdin killing of bacteria, the magnitude of its importance is greatly influenced by the strain type and the peptide sequence. Similarly, while in general an aliphatic side chain is functionally preferred over a polar residue, it is also in a context-dependent manner.

### Positions 18 and 30.

A single variation of R18H converts Crp2 to Crp7 and Crp3 to Crp8. This change enhances Crp7 killing of both E. coli and S. aureus compared to Crp2, resulting in a factor of 2 improvement in vLD90, vLD99, and vLD99.9 for E. coli and a significant but less improvement for S. aureus. At high concentrations this functional improvement is even more pronounced (see Fig. S7A and B). In contrast, Crp3 and Crp8 are essentially functionally equivalent in their killing of both strains (see Fig. S7C and D). A single amino acid change, Y30H, also differentiates the Crp2/Crp14 pair, where the His residue is much more active as compared to Tyr which can be seen in the reduction in vLD values by ~2-4-fold for E. coli and by close to 2-fold for S. aureus (see Fig. S7E and F). These results suggest that His may be superior to either cationic or hydrophobic residues in cryptdins at certain positions.

### Positions 19 and 25.

The M19I and K25R variations, each occurring once, differentiate the Crp9/Crp15 and Crp8/Crp13 pairs, respectively. Met is moderately less hydrophobic than Ile according to the Wimley-White whole residue hydrophobicity scales (68), and the M19I variation increases the vLD values of the Crp9/Crp15 pair by approximately a factor of 2 with respect to E. coli killing and marginally for S. aureus indicative of a preference of Met over Ile (see Fig. S8A and B). In contrast, the charge neutral variation, K25R, is largely inconsequential with respect to cryptdin killing of both strains by Crp8/Crp13. Arg in Crp13 is slightly preferred over Lys in Crp8 with respect to the killing of E. coli (see Fig. S8C). However, at high concentrations Crp8 is more active than Crp13 against S. aureus (see Fig. S8D), suggesting a preference of Lys over Arg at position 25. The lack of a clear preference for either cationic residue may reflect the fact that both Arg and Lys residues are heavily present in cryptdins, which is in sharp contrast to what is seen in human defensins where only Arg is found (69).

### Positions 29 and 31.

In contrast to the M19I variation found in the Crp9/Crp15 pair, the L29M variation in the Crp1/Crp16 and Crp17/Crp8 pairs is functionally neutral with the exception that Leu29 in Crp1 is preferred over Met29 in Crp16 at high concentrations against S. aureus (see Fig. S9A to D). The L29M variation increases the vLD99.9 value by 40% (from 11.9 to 17.0 μg/mL) for the Crp1/Crp16 killing of S. aureus. Notably, while the T31M variation is slightly deleterious with respect to the Crp1/Crp11 killing of E. coli (see Fig. S9E), it is detrimental with respect to Crp1/Crp11 killing of S. aureus (see Fig. S9F), as can be seen by the 2-fold increase in vLD values. Clearly, Met at position 31 in Crp11 is disfavored over Thr in Crp1 at the same position.

### The G15R variation.

**FIG 8:**
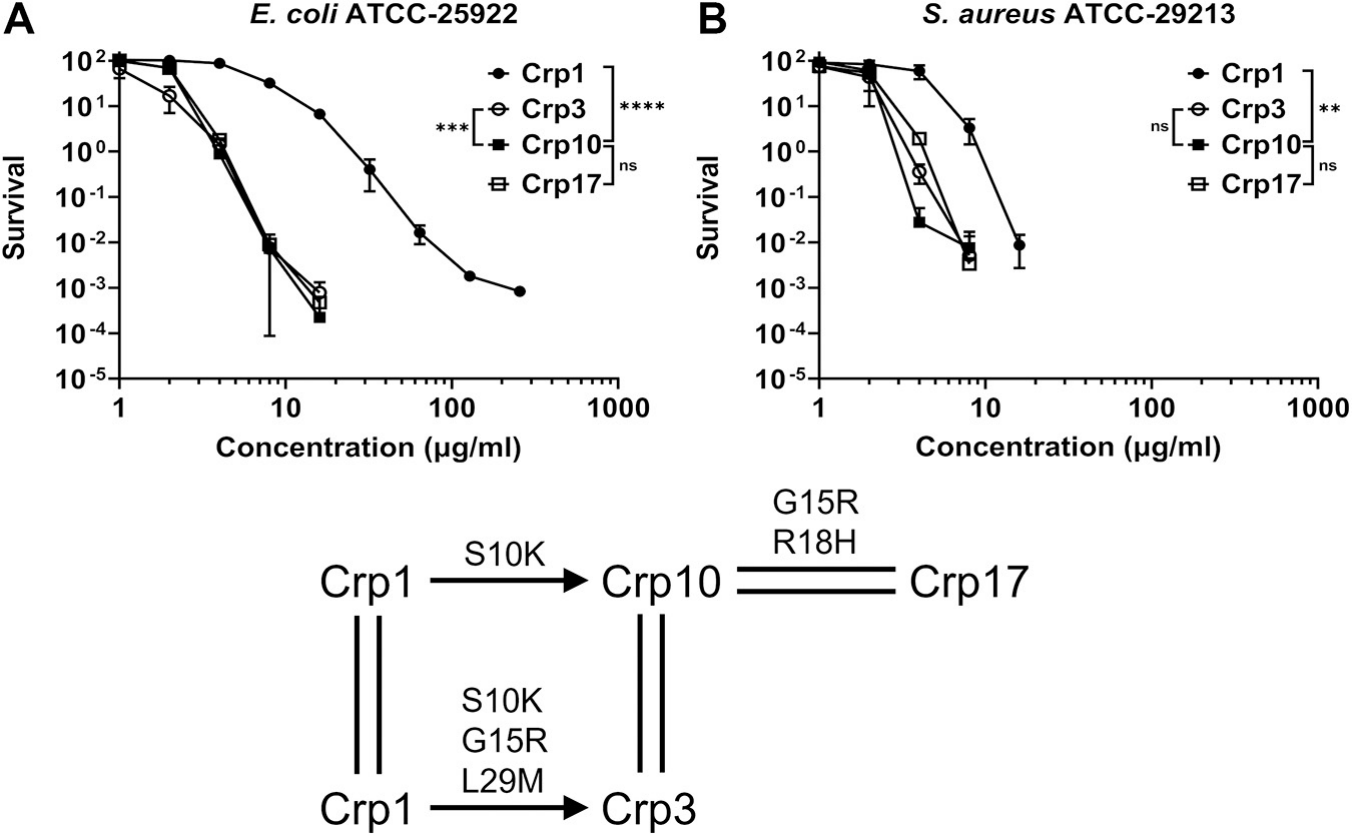
Functional impartation of the G15R variation aided by additivity cycles. (A) Survival curves of E. coli ATCC 25922 in the presence of Crp1, Crp3, Crp10, and Crp17. (B) Survival curves of S. aureus ATCC 29213 in the presence of Crp1, Crp3, Crp10, and Crp17. Since Crp3 and Crp10 are functionally “equivalent,” the two variations G15R and L29M are presumably “inconsequential.” A similar conclusion can be drawn for the G15R and R18H variations. Statistical significance was determined by using a two-way ANOVA test (*, *P* < 0.05; **, *P* < 0.01; ***, *P* < 0.001; ****, *P* < 0.0001).

Among the 17 cryptdins, Arg appears 11 times at position 15 and Gly six times. Although no direct pairwise comparison is present to permit the dissection of the mutational effect of G15R, an additivity loop can be constructed to extract it from the data from Crp1, Crp3 and Crp10 (Fig. 8). The single variation S10K (from Crp1 to Crp10) significantly improved cryptdin killing of both E. coli and S. aureus as was previously mentioned (Fig. 7A and B). Three variations, S10K, G15R and L29M, convert Crp1 to Crp3. Since Crp3 is functionally equivalent to Crp10 (Fig. 8) and the L29M variation is inconsequential in Crp1 (see Fig. S9A to D), the G15R variation likely has little impact on the bactericidal activity (70, 71). A similar conclusion can be drawn from the comparison of Crp10 with Crp17, where two variations to Crp10, G15R and R18H, yield Crp17 (Fig. 8). As was noted earlier, the R18H variation either enhances (in the Crp2/Crp7 pair) or slightly reduces (in the Crp3/Crp8 pair) cryptdin killing of E. coli and S. aureus (see Fig. S7A to D). The fact that Crp17 is largely equivalent to Crp10 suggests that the mutational effect of G15R is likely minimal.

### Functional effects of disulfide bonding in cryptdins.

**FIG 9:**
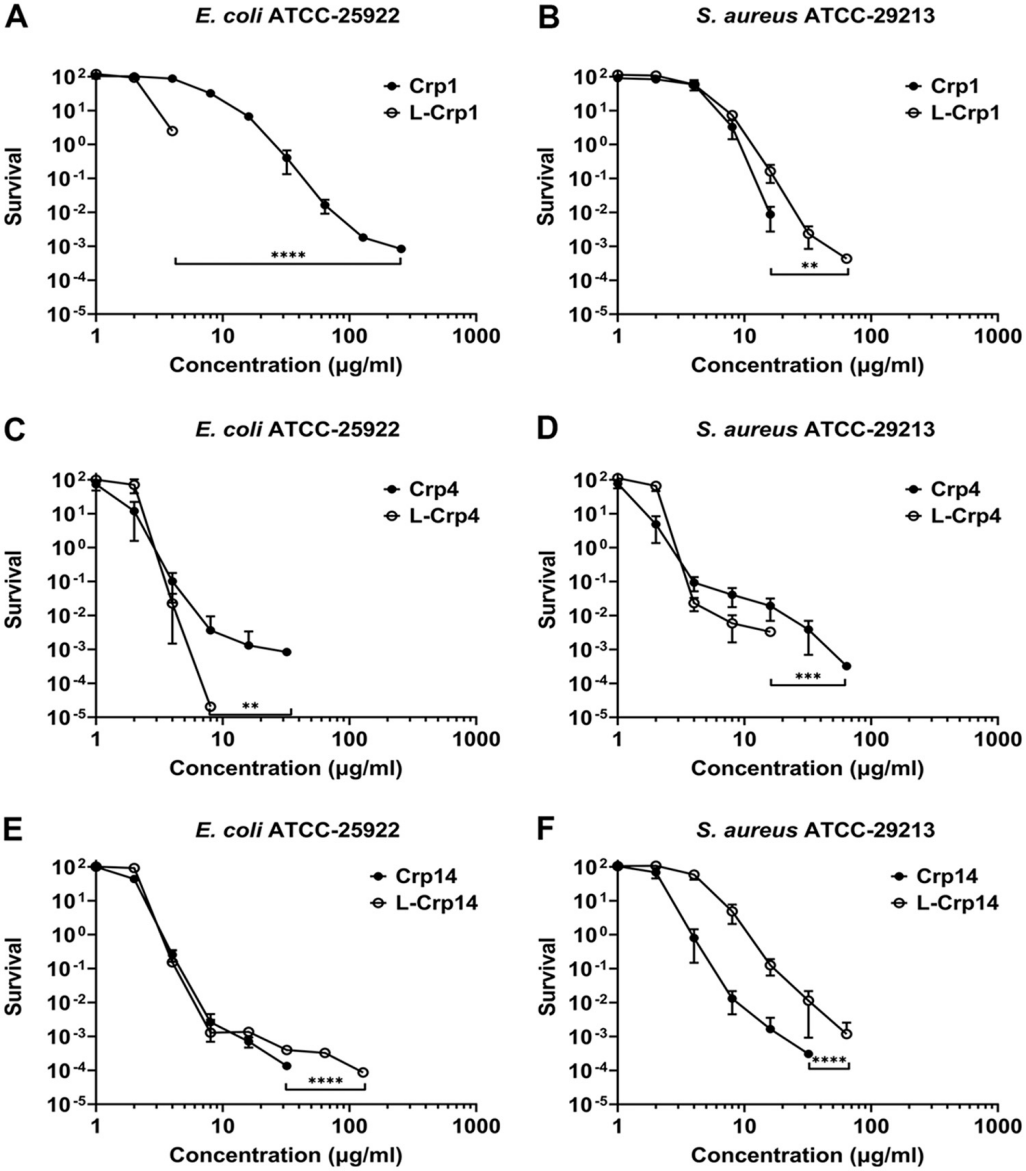
Effects of disulfide bonding in cryptdins on their antibacterial activity. (A and B) Survival curves of E. coli ATCC 25922 (A) and S. aureus ATCC 29213 (B) exposed to Crp1 and its linear form L-Crp1. (C and D) Survival curves of E. coli ATCC 25922 (C) and S. aureus ATCC 29213 (D) exposed to Crp4 and its linear form L-Crp4. (E and F) Survival curves of E. coli ATCC 25922 (E) and S. aureus ATCC 29213 (F) exposed to Crp14 and its linear form L-Crp14. Each curve is the mean of triplicate experiments. Statistical significance was determined by using a two-way ANOVA test (*, *P* < 0.05; **, *P* < 0.01; ***, *P* < 0.001; ****, *P* < 0.0001).

Published studies give a variety of accounts for the functional ramification of disulfide bonds in human defensins, ranging from a reduction, no change, or an enhancement in bactericidal activity often in a species-specific fashion (72–75). Much of the previous SAR studies on cryptdins has focused on the reduced version of Crp4 (52, 54, 55, 58) which, in general, has exhibited a bactericidal activity equivalent to or greater than that of its natively folded form. To better understand how the three invariant disulfide bonds in cryptdins impact their bactericidal activity, we selected three cryptdins, Crp1, Crp4, and Crp14, and synthesized their corresponding linear isoforms in which the six Cys residues are all replaced by Ala. As shown in Table 1 and Fig. 9A, the loss of disulfide bonds in Crp1 dramatically enhanced its killing of E. coli. While wild-type Crp1 at 256 μg/mL reduced E. coli survival by 5 orders of magnitude, linear Crp1 completely decimated it at 8 μg/mL. In fact, linearization of Crp1, one of the weakest cryptdins, made it into one of the most potent against E. coli. In contrast, the disulfide bonds are important for the Crp1 killing of S. aureus, particularly at high concentrations where the elimination of S. aureus growth was only achieved by linear Crp1 at 128 μg/mL in contrast to wild-type Crp1 where it was achieved at 32 μg/mL (Fig. 9B).

The loss of disulfide bonds in Crp4 had little effect on its killing of both E. coli and S. aureus in the low concentration range (Table 1 and Fig. 9C and D) since the vLD values of Crp4 and of linear Crp4 were highly similar. However, the bactericidal activity of wild-type Crp4 at higher concentrations is noticeably attenuated compared to its linear counterpart. In fact, linear Crp4 at 8 μg/mL reduced E. coli survival by nearly 7 orders of magnitude compared to wild-type Crp4 at 32 μg/mL, which achieved a reduction in survival of only 5 orders of magnitude. Similarly, complete inhibition of S. aureus growth was attained with linear Crp4 at 32 μg/mL and with wild type Crp4 at 128 μg/mL.

Crp14 differs from either Crp1 or Crp4 in that wild-type Crp14 and its linear form display nearly superimposable survival curves for E. coli for concentrations up to 32 μg/mL (Fig. 9E). At higher concentrations, however, the former becomes more active indicative of the importance of the disulfide bonds in Crp14 for the killing of E. coli. The importance was more evident with respect to the killing of S. aureus (Fig. 9F), where the loss of disulfide bonds increased the vLD values of Crp14 by 2- to 3-fold. Taken together, these data indicate that the functional implication of the disulfide bonds in cryptdins is dependent both upon the peptide sequence and the species of the bacteria, likely reflecting the mechanistic complexity of defensin killing of bacteria.

### Self-association of cryptdins in solution and functional implication. 

Growing evidence suggests that the ability of α-defensins to self-associate and to dimerize, oligomerize and multimerize upon target binding contributes to their functional versatility and mechanistic complexity (16, 76, 77). Although Crp14 crystallized as a dimer at a concentration of 14 mg/mL (pH 4.6), whether it is capable of dimerizing at lower concentrations and/or physiological pH is unclear, nor has the functional consequence of cryptdin dimerization yet been determined.

**FIG 10:**
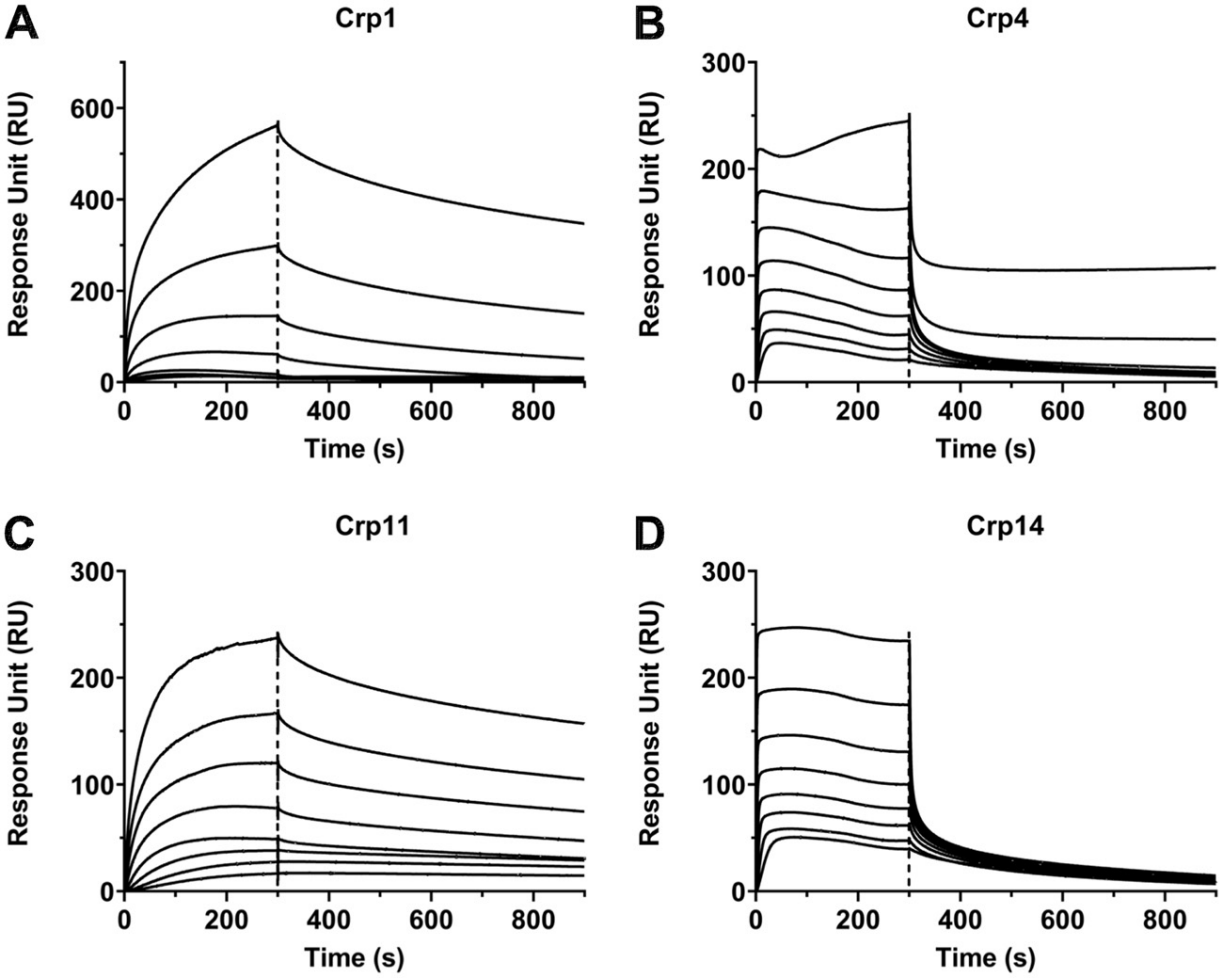
Representative association and dissociation kinetics of cryptdins analyzed by surface plasmon resonance. Cryptdins in a 2-fold dilution series at concentrations ranging from 15.625 to 2000 nM were analyzed on self-immobilized sensor chips with resonance units (RUs) of 404 for Crp1 (A), 541 for Crp4 (B), 458 for Crp11 (C), 488 for Crp14 (D). To suppress nonspecific electrostatic interactions, an additional 150 mM NaCl was added to the running buffer for Crp1 and Crp11.

We examined Crp1, Crp4, Crp11, and Crp14 for their ability to self-associate at 25°C in 10 mM HEPES, 150 mM NaCl, 3 mM EDTA, 0.005% surfactant P20 (pH 7.4) using surface plasmon resonance (SPR) techniques. As shown in Fig. 10, two distinctive self-association/dissociation isotherms were observed as increasing concentrations of cryptdin (0, 15.6, 31.3, 62.5, 125, 250, 500, 1,000, and 2,000 nM) were injected onto 400 to 500 response units of peptide immobilized on a CM5 sensor chip: (i) slow association, followed by slow dissociation kinetics for Crp1 and Crp11, and (ii) fast association, followed by fast dissociation kinetics for Crp4 and Crp14. Due to the heterogenetic nature of defensin self-association and the lack of an appropriate mathematic model for data analysis, an accurate measurement of thermodynamic parameters such as *K_d_* was unattainable. Nevertheless, since the *K_d_* values for macromolecular interactions are generally dictated by the dissociation kinetics, the SPR data indicate that Crp1 and Crp11 have a much stronger tendency to self-associate in solution than Crp4 and Crp14.

**FIG 11:**
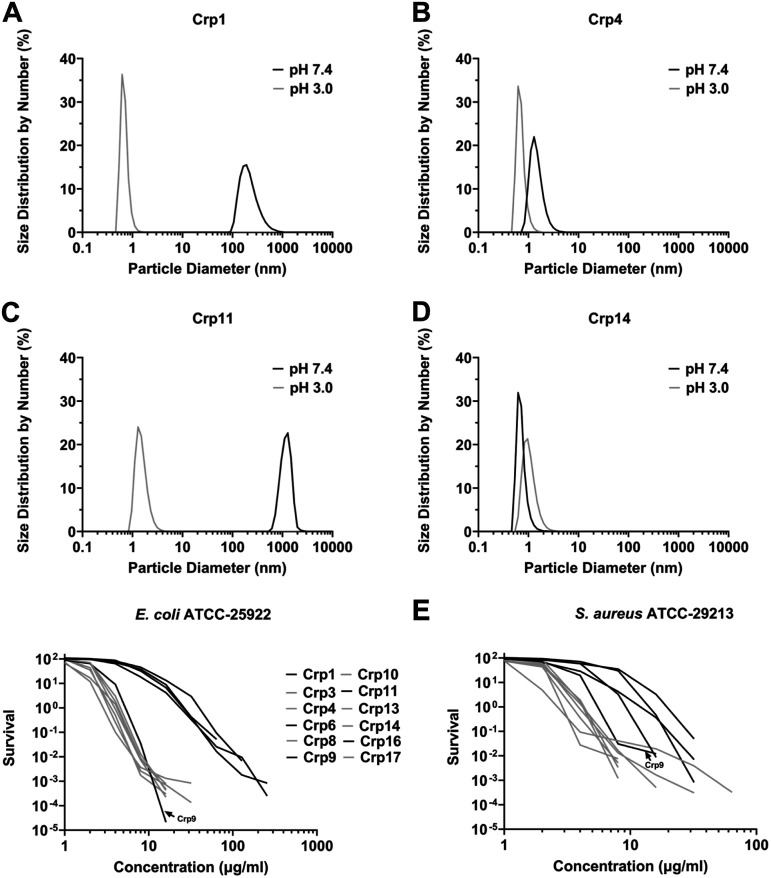
Representative particle size distributions of cryptdins analyzed by DLS. The DLS data on Crp1 (A), Crp4 (B), Crp11 (C), and Crp14 (D) are presented as gray curves (pH 3.0) and black curves (pH 7.4). The remaining DLS data on Crp3, Crp6, Crp8, Crp9, Crp10, Crp13, Crp16, and Crp17 are shown in Fig. S6. (E) E. coli and S. aureus survival curves in the presence of these 12 cryptdins are extracted from Fig. 6 with Crp1, Crp6, Crp9, Crp11, and Crp16 traced in black and Crp3, Crp4, Crp8, Crp10, Crp13, Crp14, and Crp17 traced in gray.

For verification, we subjected the four cryptdin peptides at 10 μM to dynamic light scattering (DLS) analysis in phosphate saline buffers at pH 7.4 and pH 3.0. As shown in Fig. 11A to D, all four cryptdin peptides likely existed in solution as a monomer at pH 3.0, as indicated by a particle size in diameter around 1 nm. At neutral pH, the particle sizes of Crp4 and Crp14 largely remained the same, suggesting a lack of self-association in solution. However, the particle sizes of Crp1 and Crp11 shifted dramatically at the elevated pH, to ~200 nm and >1,000 nm, respectively, indicative of formation of high-ordered soluble aggregates in solution. These DLS data are in qualitative agreement with the SPR findings.

Of note, Cryp1 and Cryp11 are among the weakest cryptdins against E. coli and S. aureus, whereas Cryp4 and Cryp14 are potently bactericidal. This phenomenon raised an intriguing possibility that the ability of cryptdins to self-associate in solution could perhaps inform their antibacterial activity. To test this hypothesis, we carried out DSL analyses on additional cryptdins at pH 3.0 and pH 7.4, including Crp6 and Crp16 (among the least active ones) and Crp3, Crp8, Crp9, Crp10, Crp13, and Crp17 (among the most active ones) (Table 1 and Fig. 11E). As shown in Fig. S10, the particle sizes of Crp6 and Crp16 at 10 μM shifted from ~1 nm to ~1,000 nm when pH was elevated from 3.0 to 7.4, indicative of self-association in solution, thus resembling Crp1 and Crp11. In contrast, Crp3, Crp8, Crp10, Crp13, and Crp17 remained largely monomeric at both pH 3.0 and pH 7.4, similar to Crp4 and Crp14. The only anomaly was Crp9 (Table 1 and Fig. 11E), which despite its antibacterial potency does self-associate at pH 7.4. Overall, these results establish a strong negative correlation as supported by a Pearson correlation analysis (see Fig. S11), albeit imperfect, between the ability of cryptdins to self-associate in solution and their antibacterial activity *in vitro* against E. coli; however, this correlation is weaker with respect to the killing of S. aureus (Fig. 11E).

### Mutational effects at position 10 on cryptdin self-association.

**FIG 12:**
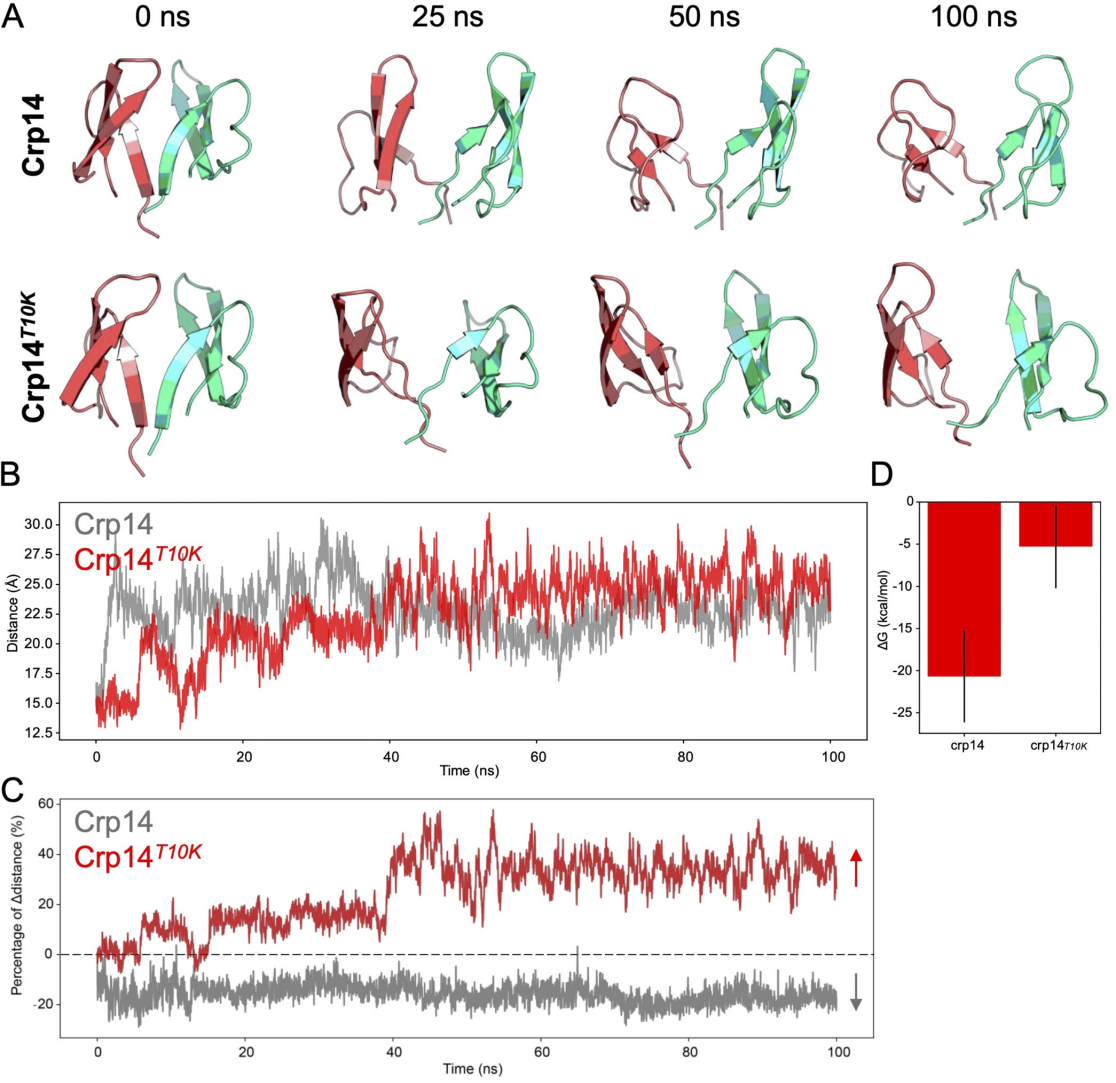
Molecular dynamics simulation of Crp14 and T10K-Crp14. (A) Snapshots of Crp14 and T10K-Crp14 dimers at different time points. (B) Distance of Cα atoms at the mutational sites between Crp14/T10K-Crp14 monomers at 100-ns trajectory. (C) Center-of-mass distance between Crp14/T10K-Crp14 monomers at 100-ns trajectory. (D) The Gibbs free energy change (Δ*G*) of Crp14/T10K-Crp14 dimers.

Lys is the most frequent amino acid residue at position 10 in cryptdins, and it is functionally important. As shown in the dimeric structure of Crp14 (see Fig. S12), the two side chains of Thr10 directly point toward each other, 8.3 Å apart between Thr10 O^γ1^ atoms, such that a T10K variation may be energetically unfavorable for cryptdin dimerization due to electrostatic repulsion. To better understand how this variation might influence cryptdin self-association and thus function, we conducted a molecular dynamics (MD) simulation study on Crp14 and its hypothetical mutant T10K-Crp14. As shown in Fig. 12A, the conformation of Crp14 was relatively stable in the 100-ns trajectory, whereas T10K-Crp14 exhibited an apparently “more loose” dimer, as measured by calculations of RMSD and radius of gyration (see Fig. S13). As expected, the distances between the Cα atoms at variation positions in the two monomers increased in the homeostatic phase of the simulation trajectory (Fig. 12B). Furthermore, the calculation of the center-of-mass distances between the two monomers showed the two monomers of T10K-Crp14 moving away from each other significantly, while the relative coordinates of wild-type Crp14 monomers remained steady (Fig. 12C). Binding free energies between the two Crp14 or T10K-Crp14 monomers were extracted from the homeostatic phase simulation trajectory. The T10K variation substantially increased the Gibbs free energy change (Δ*G*) from −20.7 ± 5.4 kcal/mol to −5.3 ± 4.9 kcal/mol (Fig. 12D), equivalent to a reduction in affinity of ~11 orders of magnitude. Of note, although the N-terminal residues (i.e., L4, V5, C6, and Y7) contributed binding free energy to cryptdin dimerization, their energetic roles were diminished by the T10K variation (see Fig. S14). Based on these results we predict that the T10K variation would probably create a considerable energetic barrier to cryptdin dimerization and/or self-association in solution. However, it is worth cautioning that MD simulations alone are not as definitive and should be taken with “a grain of salt” in the absence of mutational data.

## DISCUSSION

The antibacterial activity of human α-defensins has been extensively examined by SAR studies (16). Systematic mutational analyses have been performed on HNP1, HNP4, and HD5 (69, 76–79) and has established that C-terminal hydrophobic residues, selective Arg residues, and their ability to dimerize are the most important functional determinants of human α-defensin activity. Other studies of HNPs and HD5 have shown the molecular basis underlying the strict conservation of the nine invariant residues in mammalian α-defensins from different species. These include the salt bridge that forms between Arg5 and Glu13, Gly17, and the six Cys residues that form three structurally conserved disulfide bonds (HNP1 numbering, Fig. 1) (66, 74, 80–82). Crp4 stands out as the only cryptdin that has been extensively interrogated for its SAR with respect to its conserved salt bridge (51, 56), cationicity (53, 57, 59, 60), and disulfide bond pattern importance (52, 54, 55, 58). Our present study with the 17 cryptdin isoforms originally discovered by Ouellette and colleagues from a single jejunal crypt (37) contributes to a better understanding of how cryptdins function at the molecular level.

We have made several interesting observations. First, while Crp4 has been confirmed as being the most active cryptdin of the Crp1 to Crp6 group (37), this is only in the low concentration range of our assay. Its distinctive biphasic curves for the killing of E. coli and S. aureus indicate a significantly attenuated bactericidal activity at high peptide concentrations. Of note, markedly reduced Crp4 bactericidal activity in comparison with Crp2 and Crp3 has been reported against the facultative bacteria S. flexneri and *S.* Typhimurium under anaerobic conditions as well as the anaerobic bacterium B. fragilis (65). Second, mutational effects among the cryptdins are not only species specific but also differ significantly in magnitude from one cryptdin to another, as can be seen with the frequently occurring Lys10 residue change present in Crp3, Crp8, and Crp10. With the cases of Met at cryptdin positions 19, 29, and 31, mutational effects are also dependent on position. Third, His can be functionally superior in cryptdins to cationic residues (position 18) or hydrophobic residues (position 30). Fourth, while the functional preference for Arg or Lys in cryptdins appears to be context dependent, consistent with the previous reports (53), increased cationicity does not always lead to enhanced bactericidal activity as is seen with the G15R variation. Of note, the Ala-for-Arg substitution at the topologically equivalent position of HNP4 is highly deleterious (78). Finally, the contribution of the disulfide bonds to the antibacterial activity of cryptdins is sequence-dependent and species specific. Although we confirmed that removal of the disulfide bonds in Crp4 either had little functional impact or improved its antibacterial activity (52, 54, 55, 58), we did not fully recapitulate Crp4 results with Crp1 and Crp14. Loss of disulfide bonds in Crp1 and Crp14, while functionally detrimental to the killing of S. aureus, has the opposite effect on their killing of E. coli, a dramatic enhancement for Crp1 and a marginal reduction for Crp14. In fact, linear Crp1 is by far the strongest bactericidal peptide tested against E. coli, in sharp contrast with its parent cryptdin, which is among the weakest.

To a first approximation, the tertiary structures of cryptdins from the Crp1-like subclass appear important for the killing of S. aureus but dispensable for the killing of E. coli, suggesting that the action of cryptdins against E. coli is mechanistically distinct from that against S. aureus. Growing evidence supports the hypothesis that defensins kill S. aureus by inhibiting bacterial cell wall synthesis through sequestration of the cell wall precursor lipid II (83, 84), whereas bacterial membrane disruption by cationic antimicrobial peptides appears to be the overriding molecular and cellular event leading to E. coli death (85, 86). This is understandable as effective peptide-lipid II interactions necessitate a structured molecule to minimize the entropic penalty to achieve high affinity and strong specificity, whereas peptide-membrane interactions are of a highly fluid nature and can benefit from an unstructured peptide with maximal flexibility and the ability to make many molecular contacts. Consistent with this tenet, removal of the disulfide bonds in HNP1 and HD5, while substantially attenuating their bactericidal activity against S. aureus, is largely inconsequential with respect to their killing of E. coli (74).

An important finding from this study is that cryptdins can self-associate in solution with functional repercussion. Cryptdin-related sequence (CRS) peptides with nine Cys residues can exist as covalent homo- and heterodimers in mouse intestinal tissue with enhanced antibacterial activity compared to their monomers (87). However, this was not obvious for cryptdins. In fact, the first and only cryptdin solution structure, Crp4, was determined at pH 4.1 by NMR spectroscopy as a monomer (56, 61), contrasting with all known crystal structures of human α-defensins which were only seen as dimers (20, 21, 74, 76). (We failed to determine the solution structure of HNP1 by NMR at neutral pH where HNP1 self-associates to form soluble “aggregates” that drastically attenuates chemical shift signals.) Using SPR and DLS techniques, we demonstrated that many cryptdins at low micromolar concentrations and neutral pH, where bacterial killing assays were performed, are capable of self-association in solution. Although a monomer at 10 μM and pH 7.4, Crp14 crystallized as a dimer at 14 mg/mL and pH 4.6, underscoring the intrinsic propensity of cryptdins to dimerize at higher concentrations. Since the local concentration of cryptdins in the crypt lumen is in the range of 15 to 100 mg/mL (31), it is highly plausible that most cryptdins, if not all, exist as dimers or higher-ordered oligomers/multimers under physiological conditions.

Structural, biochemical and functional studies of obligate HNP1 and HD5 monomers have established the importance of dimerization for many, but not all, biological activities of human α-defensins. N-methylation of Ile20 of HNP1 (Fig. 5) and of Glu21 of HD5 removes two critical main-chain H-bonds at the dimer interface, preventing the two human α-defensins from forming dimers (76, 77). This backbone modification, while impairing the ability of HNP1 and HD5 to kill S. aureus and to bind or inhibit target proteins, has little impact on their bactericidal activity against E. coli, suggesting that for HNP1 and HD5 the killing of E. coli is independent of quaternary structure and, once again, mechanistically distinct from their killing of S. aureus.

Interestingly, we found a negative correlation between the ability of cryptdins to self-associate and their bactericidal activity, particularly against E. coli. In other words, monomeric cryptdins are likely more active in killing E. coli than their oligomeric or multimeric forms as indicated by our SPR, DLS, and vCC data. Although definitive evidence is lacking and could well be conferred on future studies of obligate cryptdin monomers, this correlation is generally in line with the thesis that E. coli killing by human α-defensins and cryptdins is by and large independent of both tertiary and quaternary structure.

Since cryptdin dimerization is energetically dictated by intermolecular backbone H-bonding as well as side-chain/main-chain and side-chain/side-chain interactions, the functional effect of a given variation is likely influenced, at least in part, by its impact on cryptdin self-association. What, then, influences cryptdin self-association at 10 μM and pH 7.4 as measured by DLS? Sequence comparison of all the 12 cryptdins that were subjected to DLS analysis reveals a common denominator for Crp3, Crp4, Crp8, Crp9, Crp10, Crp13, and Crp17 (with the exception of Crp14) that exist as a monomer in solution, Lys at position 10. In contrast, the four cryptdins Crp1, Crp6, Crp11, and Crp16 that self-associate in solution have either Ser or Ala at that same position. Molecular dynamics simulation studies of Crp14 and T14K-Crp14 unequivocally established that Lys10, barring other residues in cryptdins, is an important contributing factor because the electrostatic repulsion imposed by the two proximal Lys side chains is sufficient to prevent cryptdins from forming a dimer at 10 μM and pH 7.4. Since other residues also influence this process, the effect of Lys10 on cryptdin self-association is likely sequence dependent, which may explain why the magnitude of its importance at position 10 differs from cryptdin to cryptdin. By the same token, the G15R variation is functionally “silent,” presumably because it has little impact on cryptdin dimerization or self-association. Future studies using point variations and backbone-modified obligate cryptdin monomers may shed light on these important unanswered questions.

## MATERIALS AND METHODS

### Synthesis and folding of cryptdins.

Reduced cryptdin peptides 1 to 17 at 85% purity were synthesized by China Peptides (Shanghai) using Fmoc chemistry; linear Crp1, Crp4, and Crp14 with Cys-to-Ala substitutions were synthesized by GL Biochem (Shanghai) Ltd. using Fmoc chemistry. Oxidative folding of reduced cryptdins was performed, as described previously (40, 64), in 50 mM Tris-HCl buffer containing 3 mM reduced glutathione, 0.3 mM oxidized glutathione, and 1 M guanidine hydrochloride (pH 8.3). All folding reactions, monitored by analytical reversed-phase ultrahigh pressure liquid chromatography (RP-UPLC) and electrospray ionization time-of-flight (TOF) mass spectrometry, proceeded overnight at room temperature. The correctly folded products, characterized by shortened retention times and a loss of six mass units due to the formation of three disulfide bonds, were purified to homogeneity by preparative RP-HPLC. The quantification of cryptdins was done by UV measurements at 280 nm using molar extinction coefficients calculated from a published algorithm (88).

### Crystallization, data collection, and structure determination.

Crp14 was crystallized via the sitting-drop vapor-diffusion method at 20°C. Optimal crystals were obtained under the following conditions through screening and optimization: 0.1 M sodium acetate trihydrate pH 4.6 and 1.5 M ammonium sulfate. Single crystals were first washed with 5, 10, 15, 25, and 30% ethylene glycol (vol/vol) as a cryoprotectant and then flash frozen in liquid nitrogen. All data collection steps were performed on beamline BL19U1 at the Shanghai Synchrotron Radiation Facility (SSRF) using a Pilatus3 6M detector. All diffraction images were integrated, merged, and scaled using the HKL-3000 software (89). The structures were solved by molecular replacement with PHASER (90) using a truncated structure of Crp4 (PDB code 2GW9) as a starting model (56). Manual model building was performed using Coot (91), and the structures were refined with Phenix (92).

### Antibacterial activity assay.

Antimicrobial assays against *E. coli* ATCC 25922 and S. aureus ATCC 29213 were conducted using a published 96-well turbidimetric method called “virtual colony count” (67). Briefly, a 2-fold dilution series of cryptdin, ranging from 1 to 256 μg/mL in 10 mM sodium phosphate (pH 7.4), was incubated at 37°C for 2 h with E. coli or S. aureus at 1 × 10^6^ CFU/mL (a total volume of 100 μL), followed by the addition of 100 μL of twice-concentrated Mueller-Hinton broth (MHB). In addition to “negative” controls containing no cryptdin, a negative control termed “input” consisted of a portion of the same cell suspension kept on ice during the initial 2 h of incubation and added to the microplate just before addition of 100 μL of twice-concentrated MHB. Kinetic measurements of bacterial growth at 37°C were taken every 5 min over the next 12 h spectrophotometrically at 650 nm using a Tecan Spark plate reader. Data analysis utilized a Visual Basic script to automate the determination of the time necessary for each growth curve to reach a threshold change in optical density at 650 nm of 0.02 (ΔOD_650_), as previously described (67). Since the threshold times are inversely and linearly dependent on the logarithmic concentration of bacteria (67), survival data can be readily inferred from these calibration curves established in the absence of cryptdin. The rate of survival was calculated as the number of CFU of cryptdin-treated cells/number of CFU of control cells. The virtual 50% lethal dose (vLD50), vLD90, vLD99, and vLD99.9 were reported as the cryptdin concentrations that resulted in survival rates of 0.5, 0.1, 0.01, and 0.001, respectively.

### Surface plasmon resonance.

Surface plasmon resonance (SPR) experiments were performed, as described previously (80), on a BIAcore T200 system, at 25°C, in HBS-EP buffer (10 mM HEPES, 150 mM NaCl, 3 mM EDTA, 0.05% surfactant P20 [pH 7.4]), with or without additional 150 mM NaCl. Crp1 (404 RUs), Crp4 (541 RUs), Crp11 (458 RUs), and Crp14 (488 RUs), prepared in 10 mM acetate buffer (pH 5.5), were immobilized on CM5 sensor chips using the amine-coupling chemistry recommended by the manufacture. Analytes in a 2-fold dilution series at concentrations varying from 15.625 to 2,000 nM were injected into the flow cells at 30 μL/min in the running buffer, and association and dissociation assessed for 5 and 10 min, respectively. Resonance signals were corrected for nonspecific binding by subtracting the background of the control flow cell. After each analysis, the sensor chips were regenerated with 10 mM glycine (pH 1.5) and equilibrated with the running buffer before the next injection.

### Dynamic light scattering.

Dynamic light scattering (DLS) is a technique that can be used to measure the hydrodynamic diameter of proteins in solution. The principle of this technique lies in the fact that the apparent size of a protein molecule is inversely related to the speed at which it diffuses in a constant, random Brownian motion; size information can be derived mathematically through deconvolution algorithms once the rate at which the intensity of scattered laser light fluctuates as a function of time is determined by DLS (93). Cryptdins were freshly dissolved at 10 μg/mL in phosphate-buffered saline at pH 7.4 and pH 3.0, immediately filtered with a 0.22 μM disc membrane, and equilibrated for 120 s before DLS measurements at room temperature using a Malvern Zetasizer nano particle size analyzer. Plots of the relative intensity of light scattered by cryptdins versus the size were obtained. Since the scattering intensity is proportional to the square of the molecular weight (or hydrodynamic radius^6^), the intensity distribution is heavily skewed toward larger particle sizes. We presented the data in number distributions converted via Mie theory from intensity distributions using the manufacturer supplied software.

### Molecular dynamics simulation.

The three-dimension model of T10K-Crp14 dimer was constructed according to the crystal structure of wild-type Crp14 dimer using PyMOL, and fully relaxed by Rosetta package (94, 95). The models were then solvated by *LEaP* module with application of ff19SB force field (96), and subsequently submitted to MD simulations in Amber 20 (97). The trajectories were analyzed by CPPTRAJ, Gromacs 2022.1 and *MMPBSA.py* module (98), and the snapshots were visualized in PyMOL. All quantitative data in the MD simulations were analyzed and visualized by Python matplotlib package. Detailed descriptions are contained in the supplemental material.

### Statistical analysis.

Data for the pairwise comparisons shown in Fig. 7 and Fig. S6 and S9 were analyzed using a two-way analysis of variance (ANOVA) statistical test. Significance is indicated in the figures by asterisks (*, *P* < 0.05 [considered statistically significant]; **, *P* < 0.01; ***, *P* < 0.001; ****, *P* < 0.0001).

### Data availability.

The atomic coordinates and structure factors have been deposited in the Protein Data Bank (PDB accession code 7YOA).
