# Supplementary material for: Mouse α-Defensins: Structural and Functional Analysis of the 17 Cryptdin Isoforms Identified from a Single Jejunal Crypt
Source: Infect Immun. 2022 Dec 6;91(1):e00361-22. doi: 10.1128/iai.00361-22 (PMC9872612; doi:10.1128/iai.00361-22)
Supplement: Supplemental file 1 — Supplemental text, Table S1, and Fig. S1 to S14. Download iai.00361-22-s0001.pdf, PDF file, 4.1 MB [file iai.00361-22-s0001.pdf]

Supporting information

Molecular dynamics simulation. The crystal structure of wild-type Crp14 dimer has been determined in the present study, and the three-dimension model of T10K-Crp14 dimer was constructed by PyMOL mutagenesis module. The two molecular models were fully relaxed by Rosetta package to prepare the reasonable conformations for MD simulations (1,2). Then, the molecular mechanics parameters from ff19SB force field were respectively assigned to the Crp14 dimer and T10K-Crp14 dimer, which were subsequently neutralized by adding sodium/chlorine counter ions and solvated in a cuboid box of transferable interatomic potential with three points model (TIP3P) water molecules with solvent layers 12.0 Å between solute surface and box edges, by using LEaP module of Amber 20 (3). In addition, according to the volume of box, specific counts of sodium/chlorine ions were added to satisfy the 150 mM NaCl.

All MD simulations were conducted by using Amber 20 (4). The SHAKE algorithm was employed to restrict all covalent bonds involving hydrogen atoms. The pmemd.MPI module was used to perform the minimization, heat, density and equilibration of the solvated defensin systems. The solvent and ions were optimized using 5000 steps of steepest descent minimization and 5000 steps of conjugated gradient minimization with protein backbone restrained by 10 kcal $mol^{-1}Å^{-2}$. Then the systems were minimized by 5000-step steepest descent and 5000-step conjugated gradient minimizations with restrain of backbone Cα atoms by 5 kcal $mol^{-1}Å^{-2}$. Last, the systems

were fully relaxed by 15000-step of steepest descent and 15000-step conjugated gradient minimizations without any restraint, thus to remove unfavorable contacts. After minimization, the systems were heated from 0 K to 300 K in 160-ps timescale and maintained 300 K for 40 ps, by using Langevin dynamics at a constant volume, with a time step of 2 fs. A 200-ps density equilibration was then performed to optimize the heated system, which subsequently underwent two steps of NPT (T = 300 K, P = 1 atm) equilibration with or without backbone restrain for total 200 ps. Finally, the two systems were respectively submitted to a 100-ns NPT (T = 300 K, P = 1 atm) production MD simulation by using the pmemd.cuda module.

MD trajectories were analyzed by employing CPPTRAJ to extract the RMSD and radius of gyration. Conformations at different time points were exported by using Gromacs 2022.1, and visualized by PyMOL. The distances of Cα atoms of mutational sites between defensin monomers were calculated by CPPTRAJ, and the center-of-mass distances between defensin monomers were analyzed using Gromacs 2022.1. MMPBSA.py module was used to calculate the Gibbs free energy change (ΔG) between defensin monomers according to the MM/GBSA approach based on 3000 snapshots extracted from the last 60-ns trajectories, and the ΔG was further decomposed into the contributions of each residue in Crp14 or T10K-Crp14 (5). All quantitative data in MD simulations were analyzed and visualized by Python matplotlib package.

Table S1. Data collection and refinement statistics for Crp14

Resolution range, Å	27.2-1.67 (1.73-1.67)
Space group	P 65 2 2
Unit cell, Å	a=b=54.44, c=99.46
Unique reflections	10405 (826)
Multiplicity	
Completeness (%)	97.2 (80.4)
I/σ(I)	
Wilson B-factor	11.66
R-merge(I)	0.053
R-means(I)	0.054
R-pim	
CC1/2	
Reflections used in refinement	10405 (826)
Reflections used for R-free	527 (48)
R-work	0.2226 (0.2934)
R-free	0.2546 (0.2772)
Number of non-hydrogen atoms	600
Macromolecules	538
Ligands	25
Solvent	37
Protein residues	66
RMS (bond lengths), Å	0.011
RMS (bond angles), º	1.45
Ramachandran plot	
Most favored region (%)	95.16
Additional allowed (%)	4.84
Ramachandran outliers (%)	0.00
Rotamer outliers (%)	1.67
Clashscore	15.44
Average B-factor	29.12
Macromolecules	28.18
Ligands	46.08
Solvent	31.27
Number of TLS groups	2

*Statistics for the highest-resolution shell are shown in parentheses.

Figures S1-S3. The 17 cryptdin isomers, after oxidative folding and purification, analyzed by reversed phase ultrahigh pressure liquid chromatography (RP-UPLC) and electrospray ionization time-of-flight (ESI-TOF) mass spectrometry. The RP-UPLC analyses were carried out at 40 °C on an Agilent 300 SB-C18 RRHD column (1.8 µm, 2.1x50 mm) running a linear gradient of 5–65%B (solvent A, water + 0.1% TFA; solvent B, acetonitrile + 0.1% TFA) at a flow rate of 0.4 ml/min over 10 min. The determined molecular masses of cryptdins in Da are within experimental error of the theoretical values (in parentheses) calculated from their average isotopic compositions.

Figure S4. Linear Crp1, Crp4 and Crp14 analyzed by RP-UPLC and ESI-TOF mass spectrometry as described in the legend of Figs. S1-S3.

Figure S5. Superposition of two Crp14 monomers. The structural differences between the two monomers are evident at the N-terminus, where the first “visible” residue Asp3 swings by as much as 2.5 Å (Cα atoms) from one another. Also evident is the excessive movement by the side chains of Arg11 and Arg15. An asymmetric mode of dimerization of Crp14 likely induces these unequal changes in conformation to the two monomers. Prepared with the PyMOL Molecular Graphics System, Version 2.0 Schrödinger, LLC.

Figures S6-S9. Pairwise comparisons of cryptdins in the killing of *E. coli* and *S. aureus.* Each curve is the mean of triplicate experiments. These killing curves of cryptdins are

re-graphed from Figure 6. Statistical significance was determined using a two-way ANOVA test, p* < 0.05, p** < 0.01, p*** < 0.001, and p**** < 0.0001.

Figure S10. Representative particle size distributions of crypdtins analyzed by dynamic light scattering. DLS data on Crp3, Crp6, Crp8, Crp9, Crp10, Crp13, Crp16 and Crp17 are presented in grey curves (pH 3.0) and back curves (pH 7.4).

Figure S11. Pearson correlation between Crp bactericidal activity against *E. coli* and *S. aureus* (vLD90 or vLD99) and Crp self-association (log (particle size)) at pH 7.4. Crp9 was excluded from the correlation analysis. A Pearson correlation coefficient, r between 0.8 and 1.0, indicates a very strong correlation.

Figure S12. Orientation of Thr10 residues in Crp14 dimer. Prepared with the PyMOL Molecular Graphics System, Version 2.0 Schrödinger, LLC.

Figure S13. Root mean square deviations (**A**) and radius of gyration (**B**) of Crp14 or T10K-Crp14 at 100-ns trajectories.

Figure S14. Contributions of individual residues in Crp14 and T10K-Crp14 to MM/GBSA binding free energy.

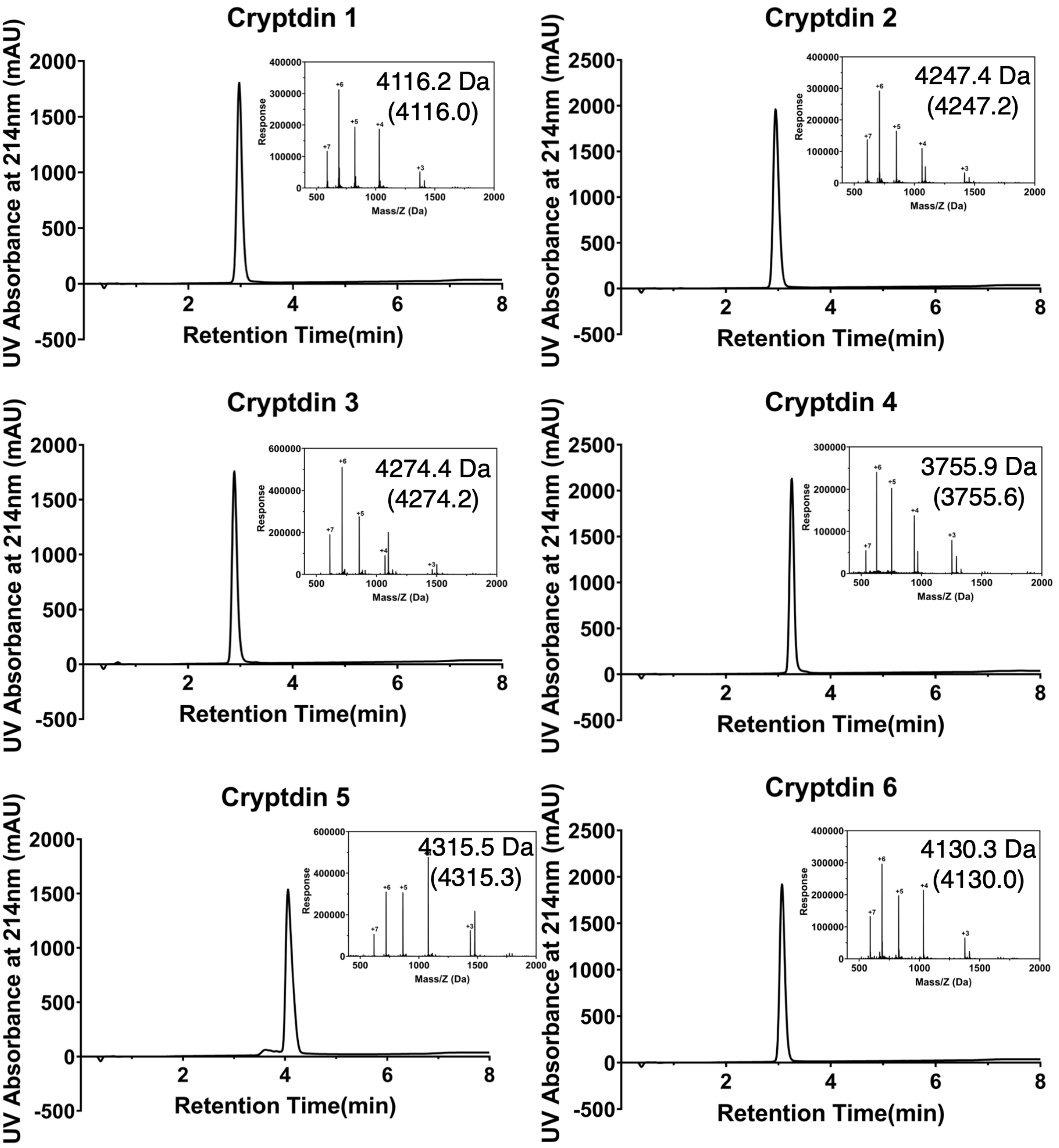
Cryptdin 1
4116.2 Da
(4116.0)
Cryptdin 2
4247.4 Da
(4247.2)
Cryptdin 3
4274.4 Da
(4274.2)
Cryptdin 4
3755.9 Da
(3755.6)
Cryptdin 5
4315.5 Da
(4315.3)
Cryptdin 6
4130.3 Da
(4130.0)
UV Absorbance at 214nm (mAU)
Retention Time(min)
Response
Mass/Z (Da)

Fig. S1

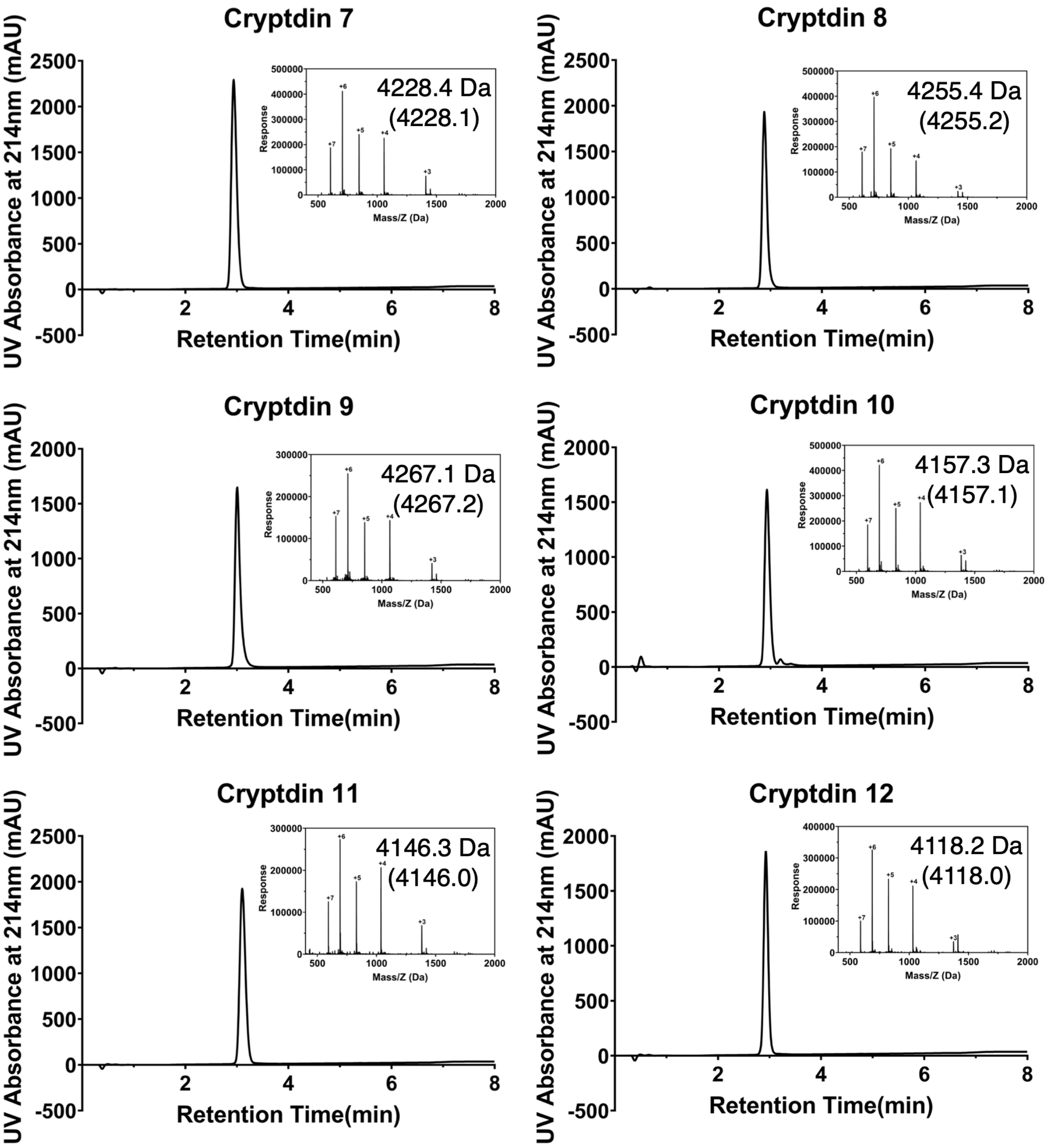
Cryptdin 7
4228.4 Da
(4228.1)
Cryptdin 8
4255.4 Da
(4255.2)
Cryptdin 9
4267.1 Da
(4267.2)
Cryptdin 10
4157.3 Da
(4157.1)
Cryptdin 11
4146.3 Da
(4146.0)
Cryptdin 12
4118.2 Da
(4118.0)
UV Absorbance at 214nm (mAU)
Retention Time(min)
Response
Mass/Z (Da)
+7
+6
+5
+4
+3

Fig. S2

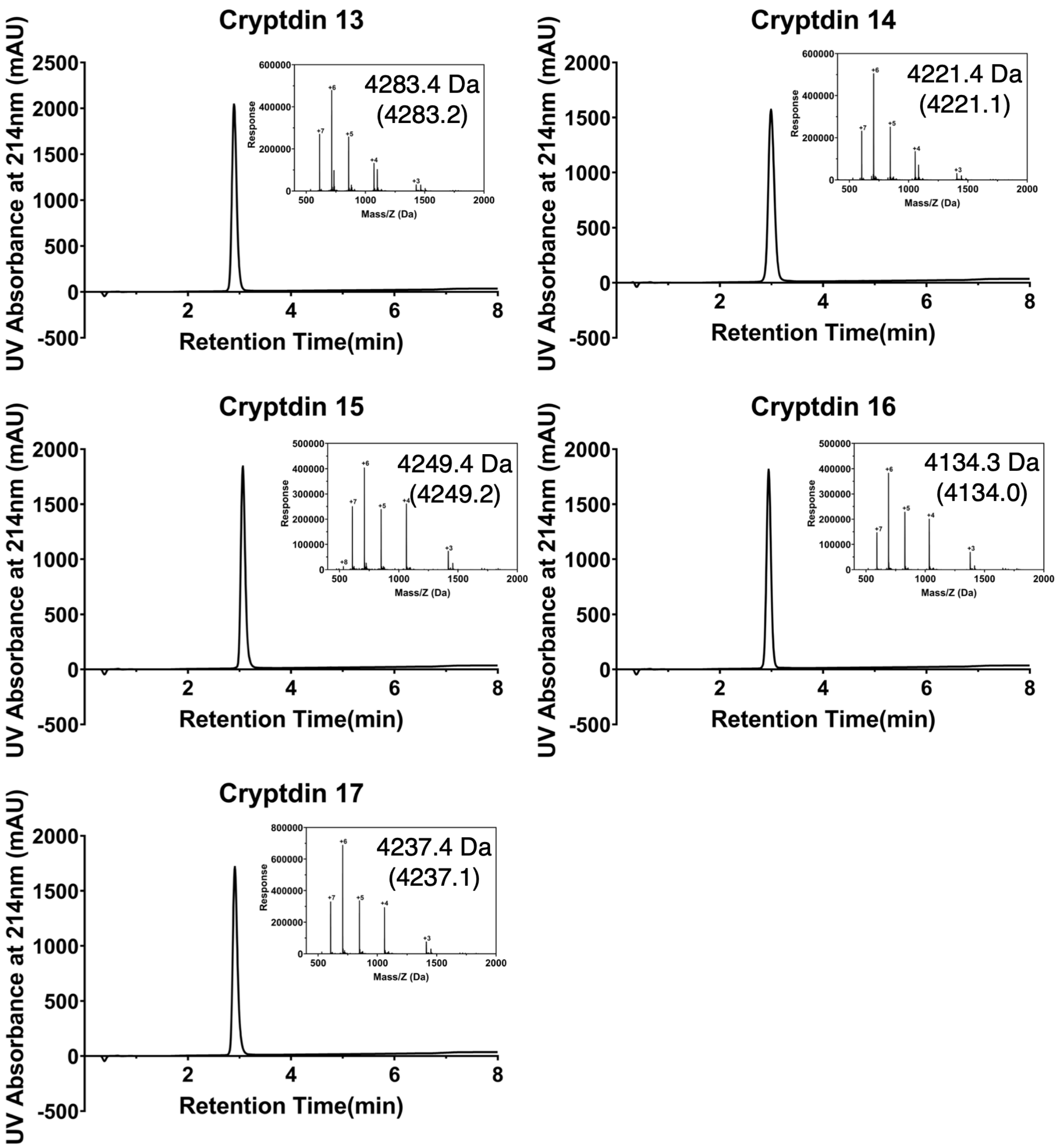
Cryptdin 13
4283.4 Da
(4283.2)
Cryptdin 14
4221.4 Da
(4221.1)
Cryptdin 15
4249.4 Da
(4249.2)
Cryptdin 16
4134.3 Da
(4134.0)
Cryptdin 17
4237.4 Da
(4237.1)
UV Absorbance at 214nm (mAU)
Retention Time(min)
Response
Mass/Z (Da)

Fig. S3

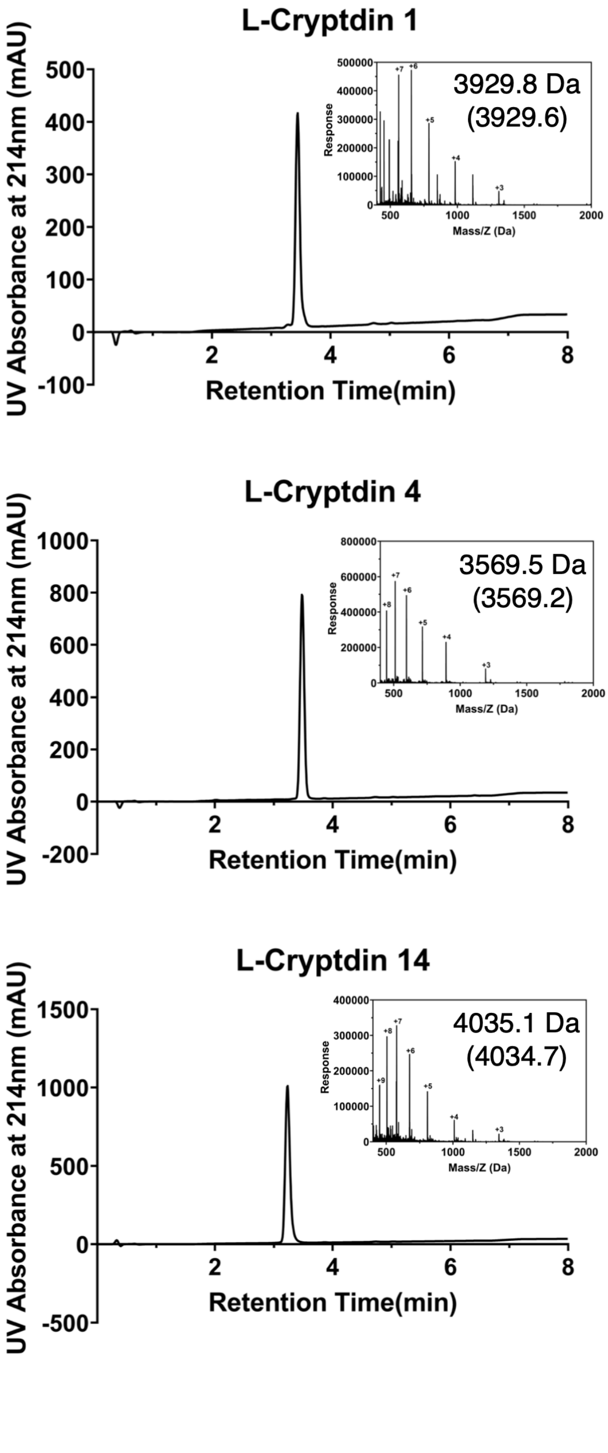

L-Cryptdin 1
3929.8 Da
(3929.6)
UV Absorbance at 214nm (mAU)
Retention Time(min)
Response
Mass/Z (Da)
L-Cryptdin 4
3569.5 Da
(3569.2)
UV Absorbance at 214nm (mAU)
Retention Time(min)
Response
Mass/Z (Da)
L-Cryptdin 14
4035.1 Da
(4034.7)
UV Absorbance at 214nm (mAU)
Retention Time(min)
Response
Mass/Z (Da)

Fig. S4

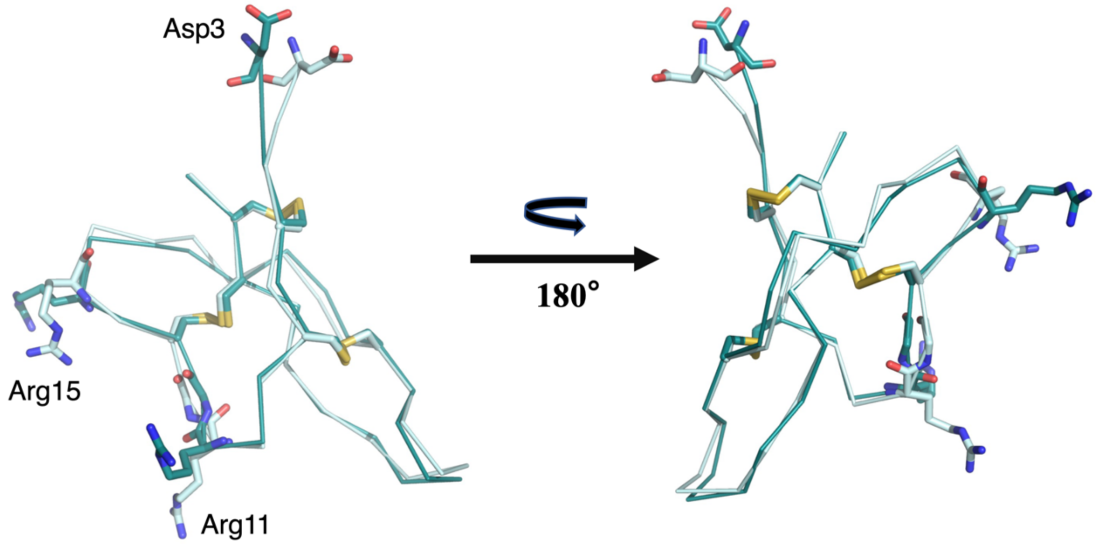

Fig. S5

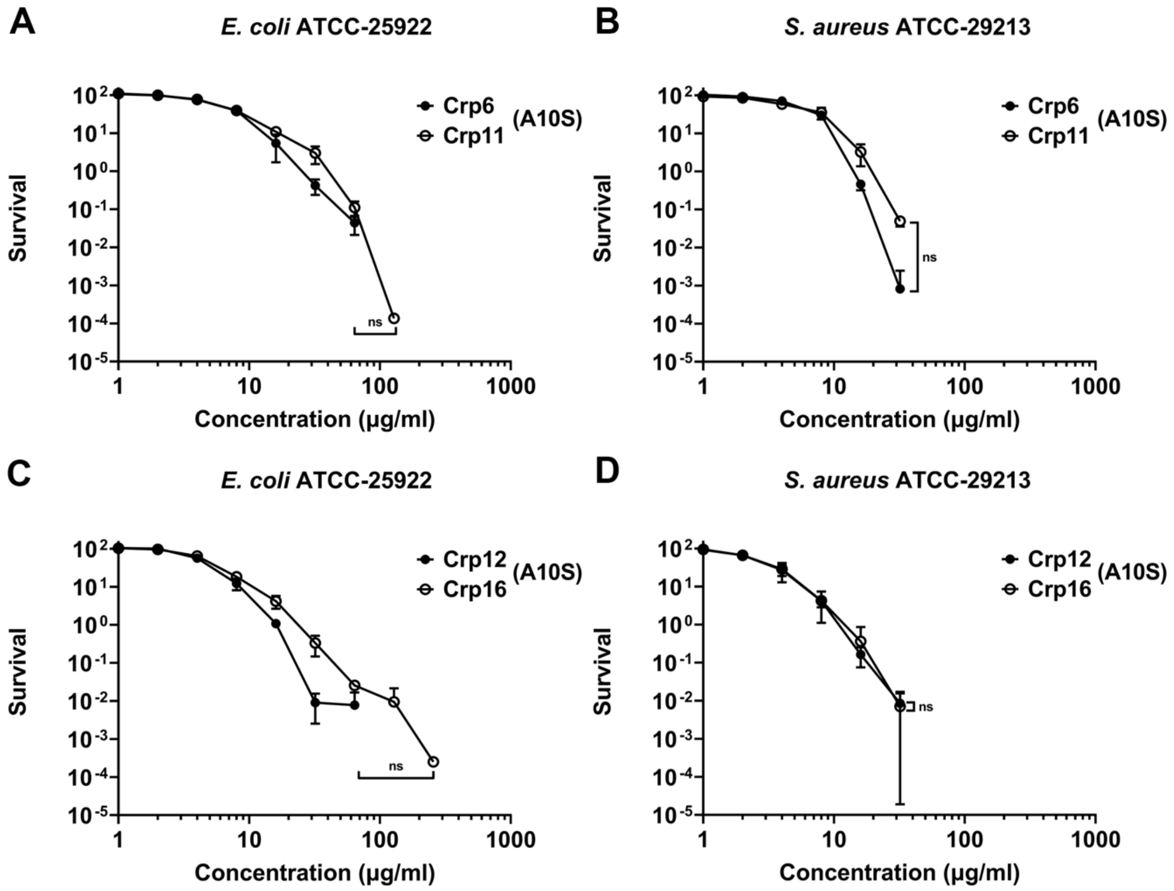
A
E. coli ATCC-25922
Crp6
Crp11
(A10S)
Survival
Concentration (μg/ml)
ns
B
S. aureus ATCC-29213
Crp6
Crp11
(A10S)
Survival
Concentration (μg/ml)
ns
C
E. coli ATCC-25922
Crp12
Crp16
(A10S)
Survival
Concentration (μg/ml)
ns
D
S. aureus ATCC-29213
Crp12
Crp16
(A10S)
Survival
Concentration (μg/ml)
ns

Fig. S6

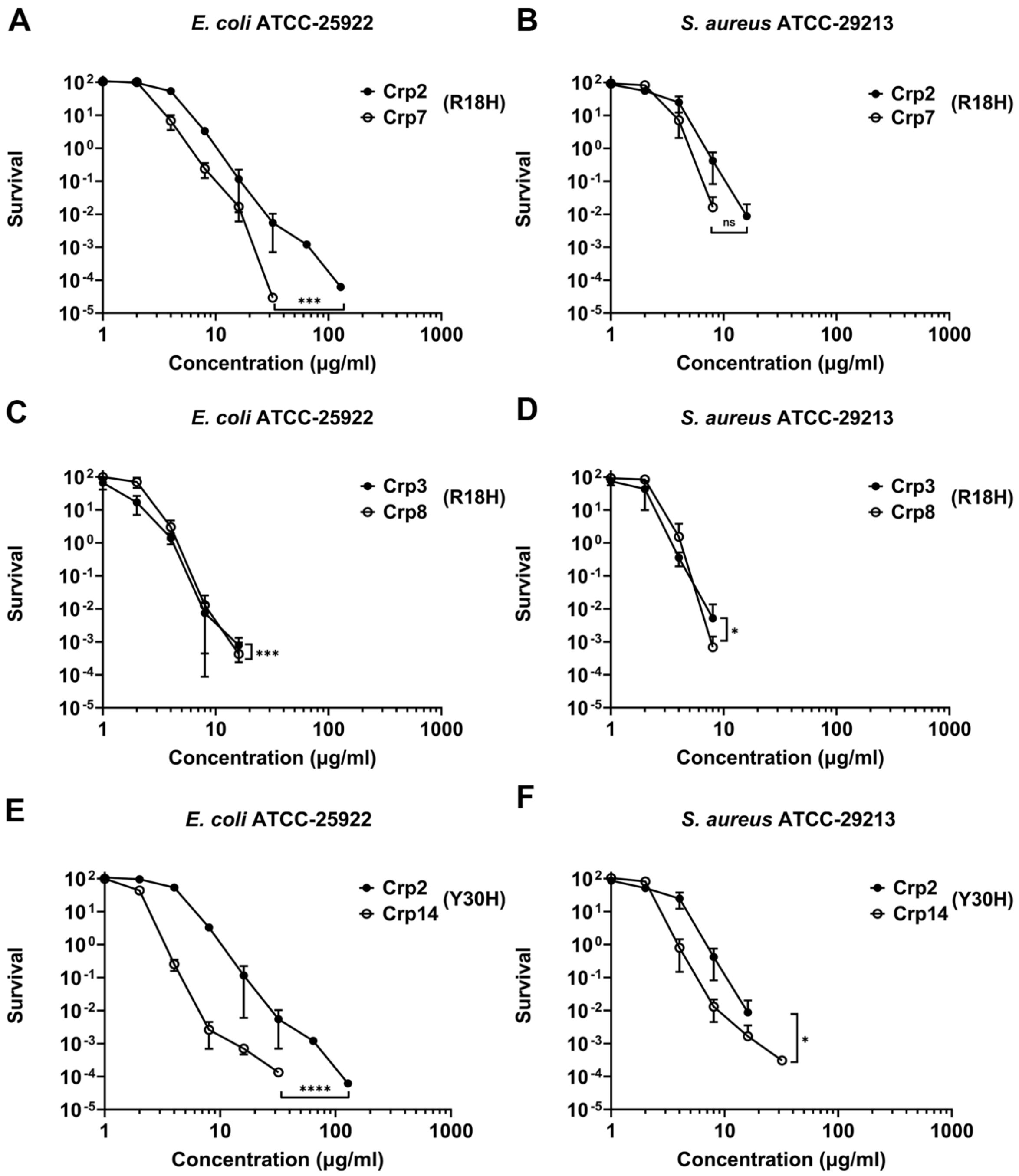
A
E. coli ATCC-25922
B
S. aureus ATCC-29213
C
E. coli ATCC-25922
D
S. aureus ATCC-29213
E
E. coli ATCC-25922
F
S. aureus ATCC-29213
Crp2
Crp7
(R18H)
Crp3
Crp8
Crp14
(Y30H)
Survival
Concentration (μg/ml)
***
ns
*
****

Fig. S7

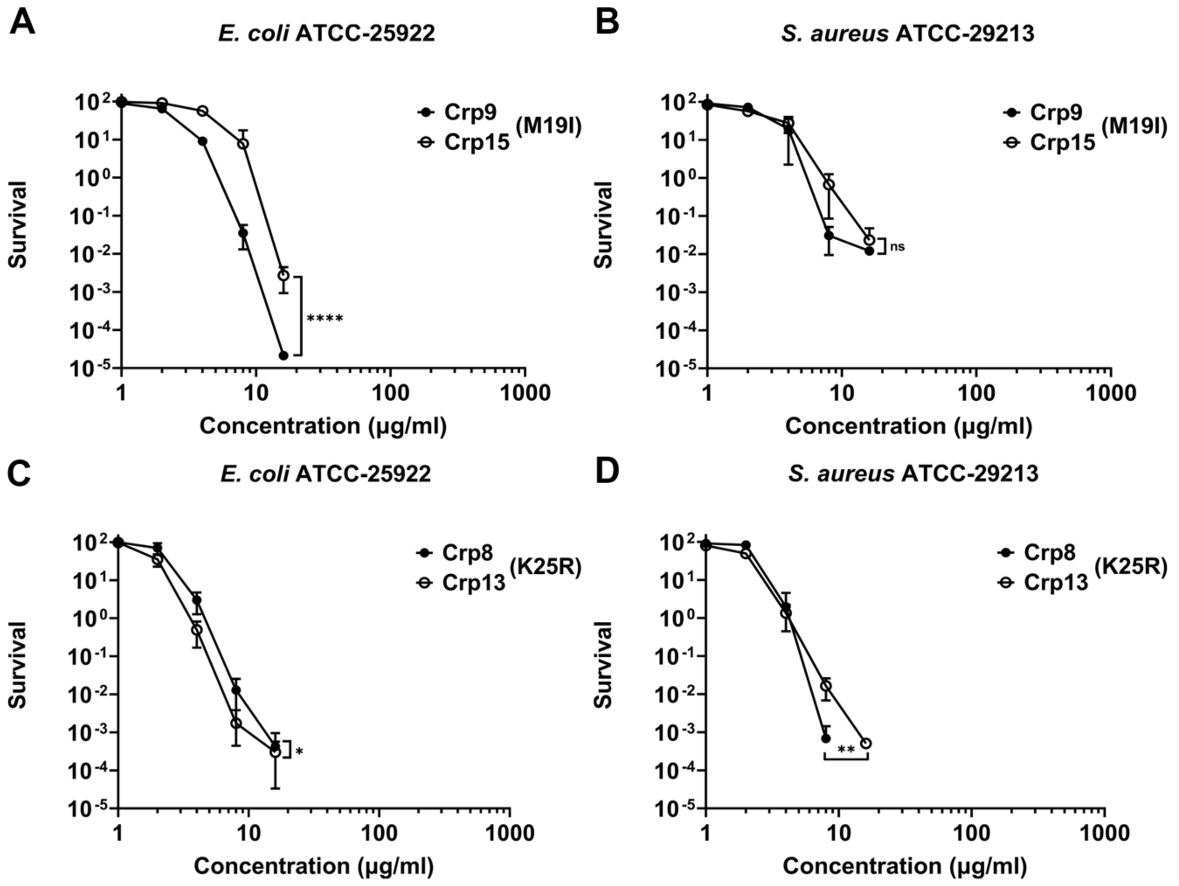
A
E. coli ATCC-25922
Crp9
Crp15 (M19I)
Survival
Concentration (µg/ml)
****
B
S. aureus ATCC-29213
Crp9
Crp15 (M19I)
ns
C
E. coli ATCC-25922
Crp8
Crp13 (K25R)
*
D
S. aureus ATCC-29213
Crp8
Crp13 (K25R)
**

Fig. S8

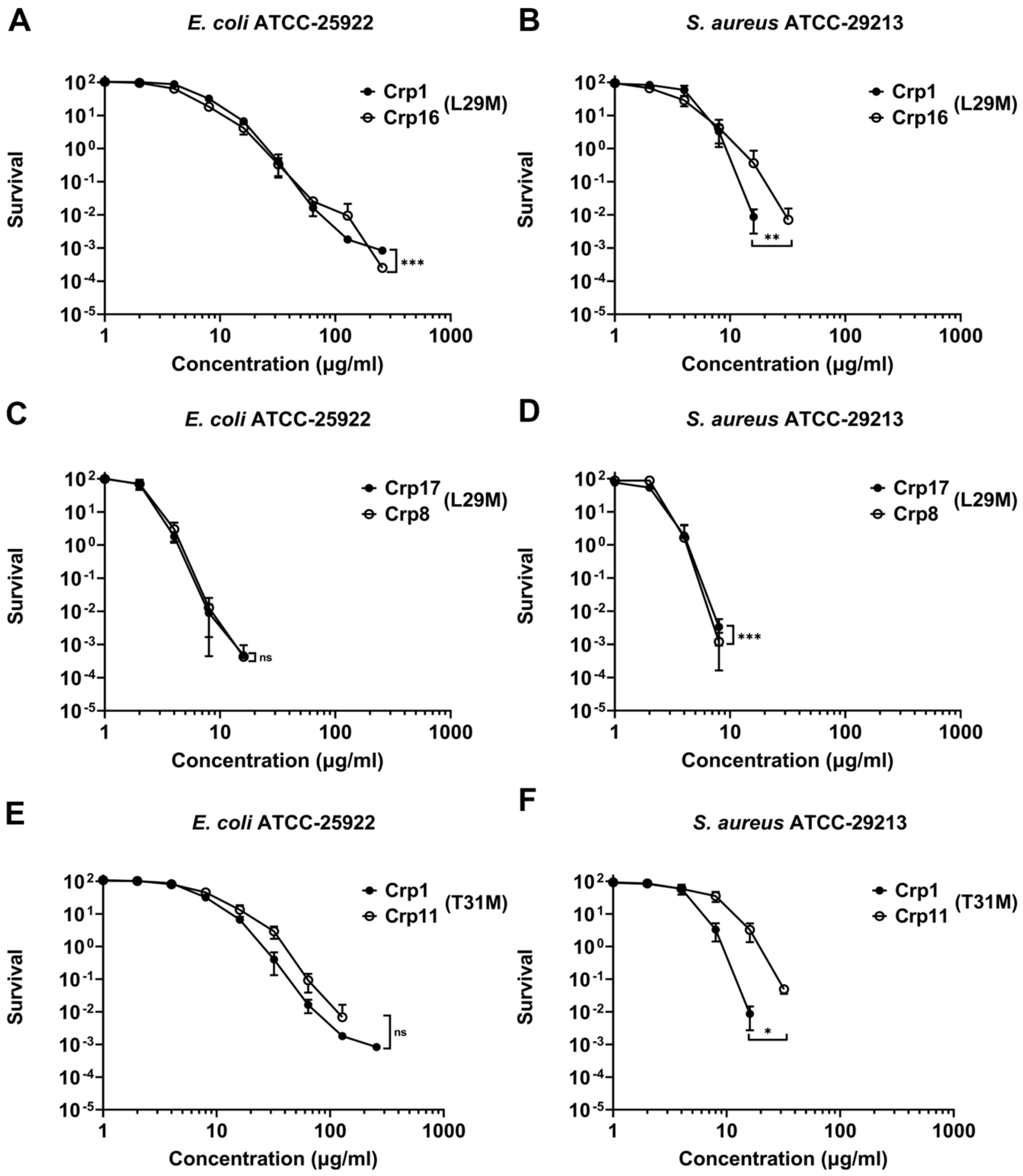

A
E. coli ATCC-25922
Crp1
Crp16
(L29M)
Survival
Concentration (μg/ml)
***
B
S. aureus ATCC-29213
Crp1
Crp16
(L29M)
**
C
E. coli ATCC-25922
Crp17
Crp8
(L29M)
ns
D
S. aureus ATCC-29213
Crp17
Crp8
(L29M)
***
E
E. coli ATCC-25922
Crp1
Crp11
(T31M)
ns
F
S. aureus ATCC-29213
Crp1
Crp11
(T31M)
*

Fig. S9

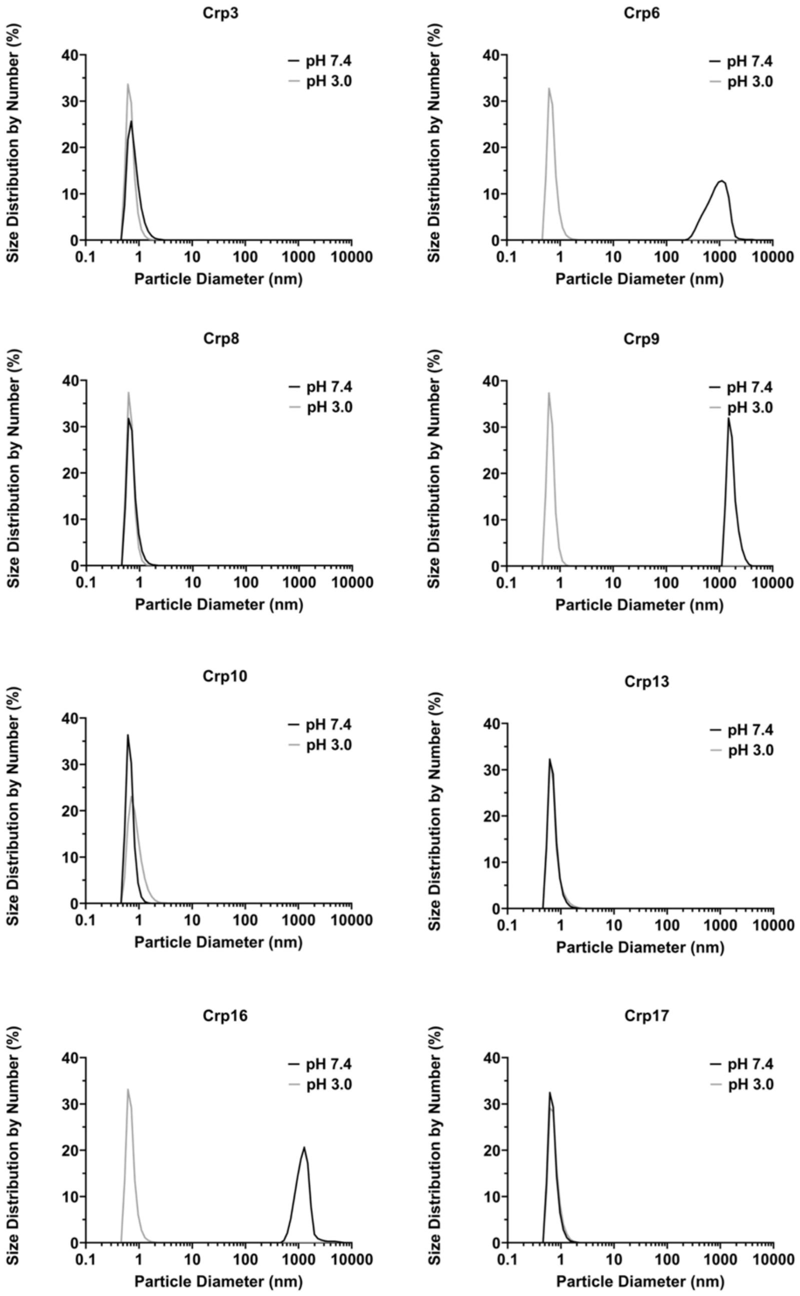
Crp3
Crp6
Crp8
Crp9
Crp10
Crp13
Crp16
Crp17
pH 7.4
pH 3.0
Size Distribution by Number (%)
Particle Diameter (nm)
0
10
20
30
40
0.1
1
10
100
1000
10000

Fig. S10

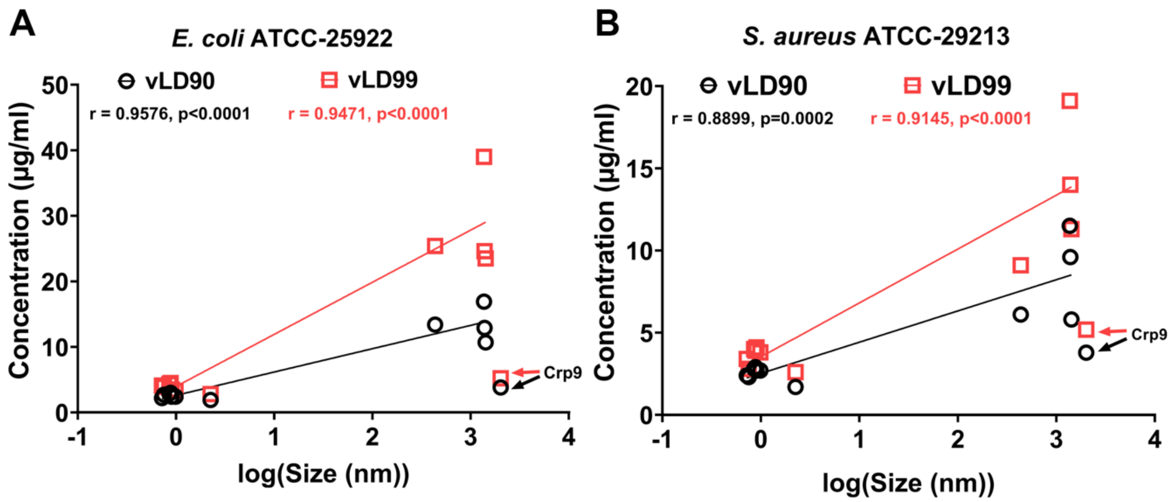
A
E. coli ATCC-25922
vLD90
vLD99
r = 0.9576, p<0.0001
r = 0.9471, p<0.0001
Concentration (µg/ml)
log(Size (nm))
Crp9
B
S. aureus ATCC-29213
vLD90
vLD99
r = 0.8899, p=0.0002
r = 0.9145, p<0.0001
Concentration (µg/ml)
log(Size (nm))
Crp9

Fig. S11

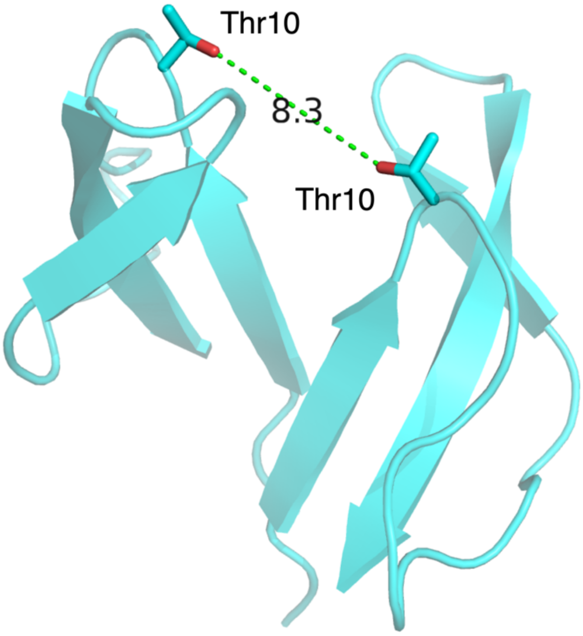

Fig. S12

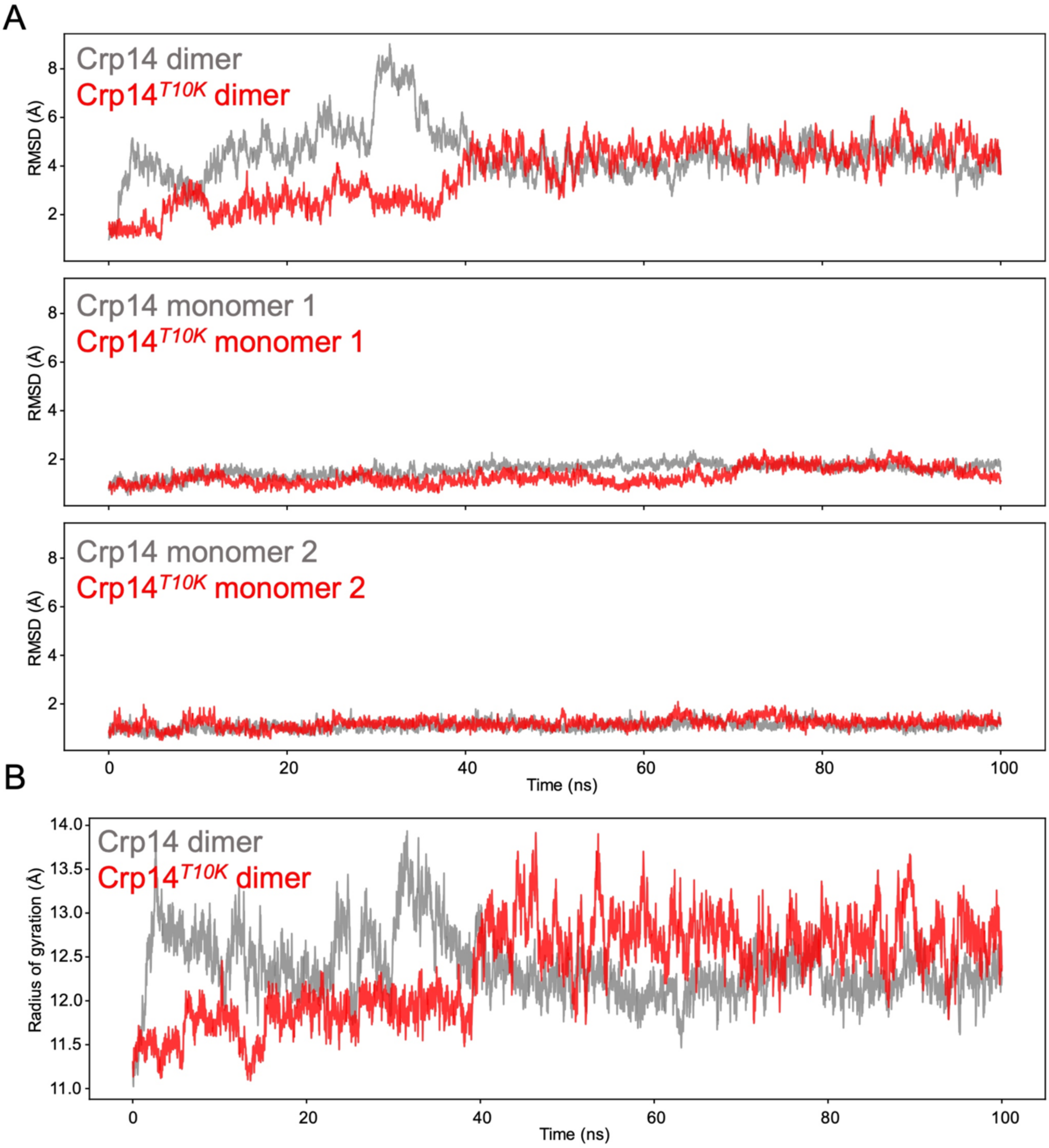
A
Crp14 dimer
Crp14T10K dimer
RMSD (Å)
Crp14 monomer 1
Crp14T10K monomer 1
Crp14 monomer 2
Crp14T10K monomer 2
Time (ns)
B
Radius of gyration (Å)
Time (ns)

Fig. S13

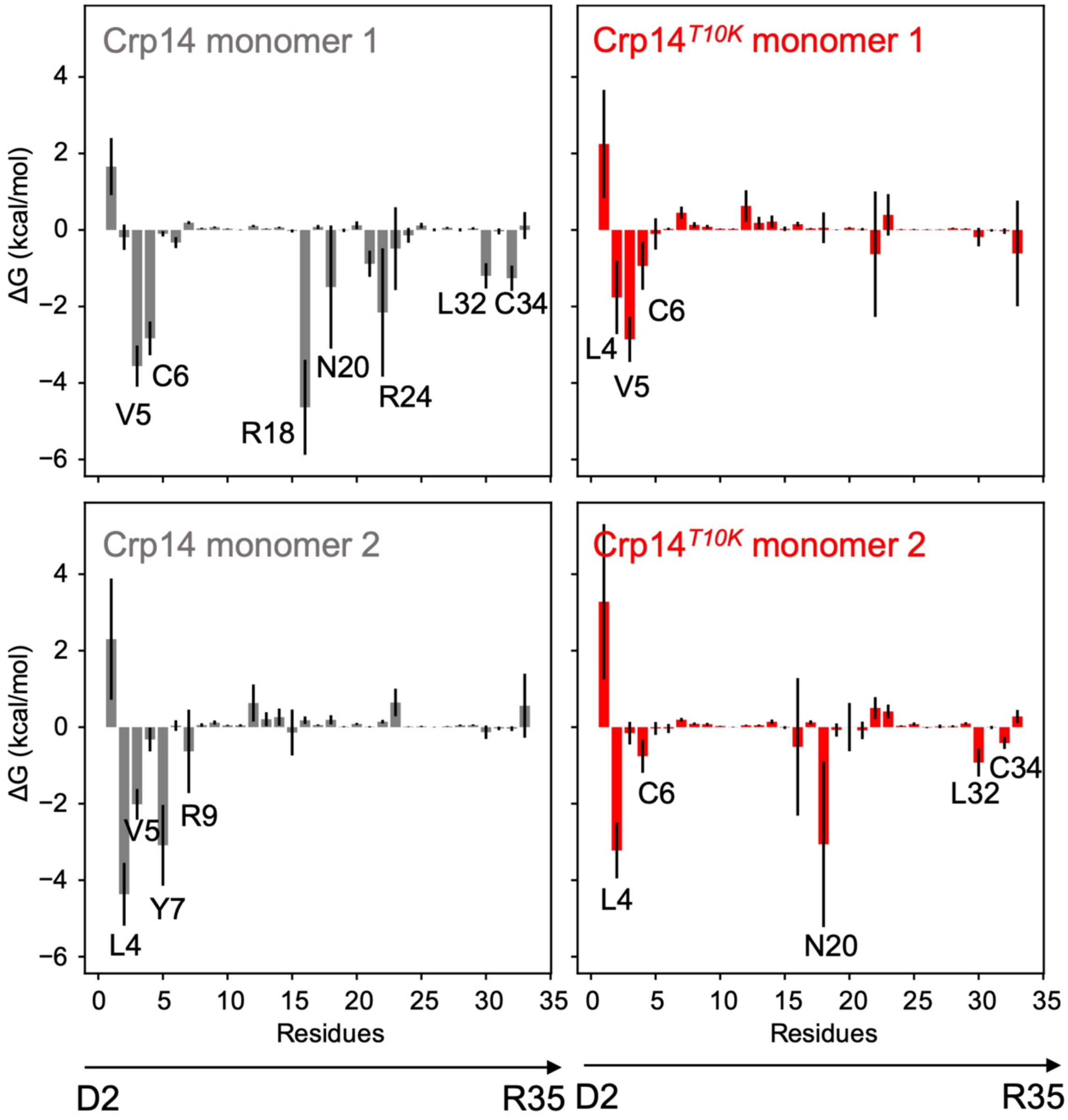

Crp14 monomer 1
Crp14^{T10K} monomer 1
Crp14 monomer 2
Crp14^{T10K} monomer 2
ΔG (kcal/mol)
ΔG (kcal/mol)
4
2
0
−2
−4
−6
V5
C6
R18
N20
R24
L32
C34
L4
V5
C6
L4
V5
R9
Y7
C6
L4
N20
L32
C34
0
5
10
15
20
25
30
35
Residues
Residues
D2
R35
D2
R35

Fig. S14

References

1. Conway, P., Tyka, M. D., DiMaio, F., Konerding, D. E., and Baker, D. (2014) Relaxation of backbone bond geometry improves protein energy landscape modeling. *Protein Sci* **23**, 47-55

2. Khatib, F., Cooper, S., Tyka, M. D., Xu, K., Makedon, I., Popovic, Z., Baker, D., and Players, F. (2011) Algorithm discovery by protein folding game players. *Proc Natl Acad Sci U S A* **108**, 18949-18953

3. Tian, C., Kasavajhala, K., Belfon, K. A. A., Raguette, L., Huang, H., Migues, A. N., Bickel, J., Wang, Y., Pincay, J., Wu, Q., and Simmerling, C. (2020) ff19SB: Amino-Acid-Specific Protein Backbone Parameters Trained against Quantum Mechanics Energy Surfaces in Solution. *J Chem Theory Comput* **16**, 528-552

4. D.A. Case, H. M. A., K. Belfon, I.Y. Ben-Shalom, J.T. Berryman, S.R. Brozell, D.S. Cerutti, T.E. Cheatham, III, G.A. Cisneros, V.W.D. Cruzeiro, T.A. Darden, R.E. Duke, G. Giambasu, M.K. Gilson, H. Gohlke, A.W. Goetz, R. Harris, S. Izadi, S.A. Izmailov, K. Kasavajhala, M.C. Kaymak, E. King, A. Kovalenko, T. Kurtzman, T.S. Lee, S. LeGrand, P. Li, C. Lin, J. Liu, T. Luchko, R. Luo, M. Machado, V. Man, M. Manathunga, K.M. Merz, Y. Miao, O. Mikhailovskii, G. Monard, H. Nguyen, K.A. O'Hearn, A. Onufriev, F. Pan, S. Pantano, R. Qi, A. Rahnamoun, D.R. Roe, A. Roitberg, C. Sagui, S. Schott-Verdugo, A. Shajan, J. Shen, C.L. Simmerling, N.R. Skrynnikov, J. Smith, J. Swails, R.C. Walker, J. Wang, J. Wang, H. Wei, R.M. Wolf, X. Wu, Y. Xiong, Y. Xue, D.M. York, S. Zhao, and P.A. Kollman. (2022) Amber 2022, University of California, San Francisco.

5. Sun, H., Li, Y., Tian, S., Xu, L., and Hou, T. (2014) Assessing the performance of MM/PBSA and MM/GBSA methods. 4. Accuracies of MM/PBSA and MM/GBSA methodologies evaluated by various simulation protocols using PDBbind data set. *Phys Chem Chem Phys* **16**, 16719-16729
